# Positive autoregulation of *Sox17* is necessary for gallbladder and extrahepatic bile duct formation

**DOI:** 10.1242/dev.203033

**Published:** 2025-01-16

**Authors:** Linh T. Trinh, Ryan R. Finnel, Anna B. Osipovich, Jessica R. Musselman, Leesa L. Sampson, Christopher V. E. Wright, Mark A. Magnuson

**Affiliations:** ^1^ Center for Stem Cell Biology, Vanderbilt University, Nashville, TN 37232, USA; ^2^ Department of Cell and Developmental Biology, Vanderbilt University, Nashville, TN 37232, USA; ^3^ Program in Developmental Biology, Vanderbilt University, Nashville, TN 37232, USA; ^4^ Department of Molecular Physiology and Biophysics, Vanderbilt University, Nashville, TN 37232, USA; ^5^ School of Arts and Sciences, Vanderbilt University, Nashville, TN 37232, USA

**Keywords:** *Sox17*, Endoderm, *Cis*-regulatory elements, Hepato-pancreato-biliary system, Mouse

## Abstract

Expression of SRY-box transcription factor 17 (*Sox17*) in the endodermal region caudal to the hepatic diverticulum during late gastrulation is necessary for hepato-pancreato-biliary system formation. Analysis of an allelic series of promoter-proximal mutations near the transcription start site (TSS) 2 of *Sox17* in mouse has revealed that gallbladder (GB) and extrahepatic bile duct (EHBD) development is exquisitely sensitive to *Sox17* expression levels. Deletion of a SOX17-binding *cis-*regulatory element in the TSS2 promoter impairs GB and EHBD development by reducing outgrowth of the nascent biliary bud. These findings reveal the existence of a SOX17-dependent autoregulatory loop that drives *Sox17* expression above a critical threshold concentration necessary for GB and EHBD development to occur, and that minor impairments in *Sox17* gene expression are sufficient to impair the expression of SOX17-regulated genes in the nascent GB and EHBD system, impairing or preventing development.

## Teaser

**Summary:** A SOX17-binding *cis*-regulatory element in transcription start site 2 of *Sox17* forms a positive autoregulatory feedback loop necessary for gallbladder and extra-hepatic bile duct formation.

## INTRODUCTION

Embryonic development and organogenesis rely on precise spatiotemporal control of gene-regulatory networks that operate in progressively complex hierarchical cascades. This precision depends largely on the synchronization of transcription factor (TF) expression and *cis*-regulatory DNA response element (CRE) binding. Understanding how specific TFs interact with distinct CREs to guide specific organ development is central to tissue and cell differentiation, as well as evolutionary divergence.

The mammalian biliary system channels, stores and releases bile produced by hepatocytes into the duodenum via the gallbladder (GB), hepatic, cystic and common ducts, and thus is essential for life (Villasenor and Stainier, 2017). Biliary atresia (BA) and other congenital anomalies of GB may cause premature death due to cholestasis, portal hypertension and cirrhosis (Alagille, 1984; Brahee and Lampl, 2022). Liver transplantation is often the only effective therapy for these developmental defects (Balakrishnan et al., 2006; Kosmidis et al., 2017; Wehrman et al., 2019; Cinalli et al., 2021).

In both mice and humans, SRY-box transcription factor 17 (*Sox17*) is necessary for endodermal hepato-pancreato-biliary (HPB) system formation and vascular-hematopoietic cell emergence (Kanai-Azuma et al., 2002; Choi et al., 2012; Villasenor and Stainier, 2017). Although *Sox17* is first expressed in all primitive and definitive endoderm, during late gastrulation expression of the gene in the foregut becomes gradually restricted to an endodermal region caudal to the hepatic diverticulum where it is essential for GB, extra-hepatic bile duct (EHBD) and ventral pancreas formation (Spence et al., 2009; Zorn and Wells, 2009).

Mounting evidence suggests that altered levels of *Sox17* expression in the gut tube or other tissues can profoundly influence developmental outcomes. In mice, reductions in *Sox17* expression in heterozygous mutants are associated with BA (Uemura et al., 2013, 2020), recapitulating reduced or absent SOX17 levels observed in the biliary epithelium of human BA patients. Furthermore, mice that are heterozygous null for *Sox17* exhibit abnormalities in both their fertility (Hirate et al., 2016) and hepatic lipid metabolism (Rommelaere et al., 2014), with these phenotypes possibly being more severe in an inbred C57BL/6 background compared to outbred CD-1 background (Choi et al., 2012).

TF dosage effects have been reported for several members of the SOX family. In human iPSC-derived chondrocytes, SOX9 variably activates different response elements depending on SOX9 levels, suggesting that subtle differences in *SOX9* expression affect craniofacial development through dosage-dependent effects on chondrocyte development (Naqvi et al., 2023). In mouse liver, *Sox9* also modulates cholangiocyte development in a dosage-dependent manner, thereby altering formation of the intrahepatic biliary system (Adams et al., 2020). Similarly, alteration in the expression of SOX2, a crucial pluripotency factor, impairs bifurcation of the esophagus and trachea in both mice and humans (Pevny and Nicolis, 2010; Abatti et al., 2023). Mice with dysregulated *Sox2* expression develop esophageal atresia with distal tracheoesophageal fistula (Abatti et al., 2023), a lethal condition wherein the trachea and esophagus fail to separate (Brunner and van Bokhoven, 2005; Que et al., 2006). These findings suggest that gene dosage effects may be a crucially important and shared feature of multiple SOX family members.

The murine *Sox17* locus contains two major promoter regions that differentially drive expression of long and short *Sox17* mRNAs, both encoding the same protein product. Transcription start site (TSS) 2 is active in both endodermal and vascular cells (Trinh et al., 2022), and a 126 bp deletion containing the TSS2 promoter (previously termed CR2) interferes with formation of both the HPB system and vascular endothelial cells (Trinh et al., 2022), causing mid-gestational developmental arrest. Conversely, deletion of the TSS1 promoter, which is preferentially expressed in vascular endothelial cells, has only minor developmental effects. However, as a 126 bp deletion of the TSS2 promoter also impairs processing of transcripts from TSS1, resulting in dual functionality of the TSS2 promoter region, we were unable to precisely delineate the role of TSS2-derived *Sox17* mRNAs during HPB organogenesis.

To overcome these experimental issues, we used CRISPR/Cas9 to mutate two CREs in the TSS2 promoter region. In doing so, we also obtained a 50 bp deletion that eliminated three CREs. Surprisingly, while the previously reported 126 bp mutation that destroys the TSS2 promoter is lethal, mice with the Δ50 bp mutation are viable, exhibit normal RNA processing from TSS1, and fail to form both a GB and cystic duct (CD). Studies of mice with these new alleles revealed that: (1) formation of the GB and EHBD system is exquisitely sensitive to *Sox17* expression dosage; (2) efficient formation of the GB and EHBD system requires multiple CREs in the promoter for TSS2; and (3) one of the CREs binds SOX17 thereby creating a positive autoregulatory feedback loop that drives *Sox17* above an expression threshold necessary for formation of the GB and EHBD system.

## RESULTS

### An allelic series of *Sox17* TSS2-adjacent promoter mutations

**Fig. 1.**
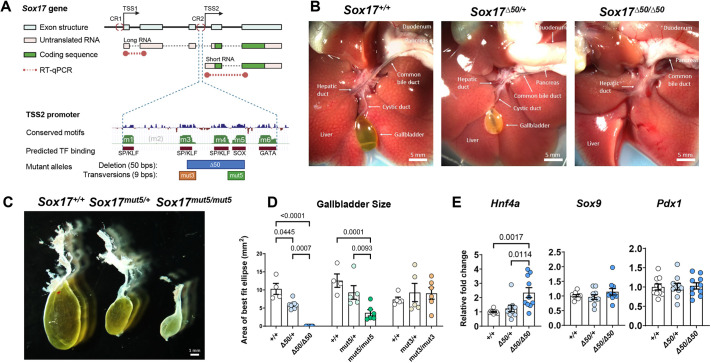
***Sox17^Δ50^* and *Sox17^mut5^* alleles impair GB formation.** (A) Schematic of *Sox17* and its two conserved promoter regions (CR1 and CR2), which drive transcription from TSS1 and TSS2, respectively. CR2 contains five discrete conserved nucleotide blocks previously referred to as m1, m3, m4, m5 and m6. Transcription factor binding corresponding to each block was predicted using JASPAR database. The location of the deletion mutation, Δ50, and the two nucleotide transversion mutations, mut3 and mut5, are indicated by colored rectangles and the exact genome coordinates can be found in the Materials and Methods. TF, transcription factor. bps, base pairs. Created in BioRender. Finnel, R. (2025) https://BioRender.com/o26n798. (B) Gross morphology analysis of the hepato-pancreato-biliary (HPB) system in adult *Sox17^+/+^*, *Sox17^Δ50/+^* and *Sox17^Δ50/Δ50^* mice showed an absence of gallbladder (GB) and cystic duct (CD) in the latter.(C) Gross morphology of dissected GBs in adult *Sox17^+/+^*, *Sox17^mut5/+^* and *Sox17^mut5/mut5^* mice showed a dose-dependent phenotype of GB hypoplasia. (D) Adult GB size in mutant and WT mice as estimated by surface area of best fitted ellipse to two dimensional images of dissected GBs. *Sox17^+/+^*, *n*=5; *Sox17*^Δ*50/+*^, *n*=9; *Sox17*^Δ*50/*Δ*50*^, *n*=8, *P*<0.0001. *Sox17^+/+^*, *n*=4; *Sox17^mut5/+^*, *n*=5; *Sox17^mut5/mut5^*, *n*=6, *P*=0.0001. *Sox17^+/+^*, *n*=5; *Sox17^mut3/+^*, *n*=5; *Sox17^mut3/mut3^*, *n*=6, *P*=0.6230. Two-way ANOVA. (E) RT-qPCR analysis of HPB gene markers in *Sox17^+/+^*, *Sox17^Δ50/+^* and *Sox17^Δ50/Δ50^* E9.5 embryos. *Hnf4a*: liver (*P*=0.0017); *Sox9:* ductal system (*P*=0.4884); *Pdx1*: pancreas and duodenum (*P*=0.9699). One-way ANOVA. *Hnf4a*: *Sox17^+/+^*, *n*=10; *Sox17*^Δ*50/+*^, *n*=9; *Sox17*^Δ*50/*Δ*50*^, *n*=10. *Sox9*, *n*=10. *Pdx1*, *n*=10. Data are mean±s.e.m.

The TSS2 promoter in *Sox17* contains five distinct evolutionarily conserved CREs (m1, m3, m4, m5, m6; Fig. 1A). Previously, we have shown that each of these elements contributes at different potencies to transcription regulation using a reporter gene assay in endodermal cells derived from mouse embryonic stem cells (mESCs), with mutations of m5 or m3 having the strongest and weakest effects on transcription, respectively (Trinh et al., 2022). To determine the function of the m5 and m3 CREs on HPB development *in vivo*, we used CRISPR/Cas9 gene editing to introduce 9 bp nucleotide transversion mutations (C↔T and G↔A), creating *Sox17^mut3^* and *Sox17^mut5^* alleles. In doing so, a fortuitous microhomology-mediated end joining event also resulted in a 50 bp deletion mutation spanning most of the m3-m4-m5 region, hereafter referred to as *Sox17^Δ50^*. Based on their sequence motifs, the m3 and m4 CREs likely bind SP or KLF (Specific Protein or Krüppel-like) factors, whereas the m5 CRE represents a predicted SOX-binding element (Fig. 1A). Contrary to the embryonic lethal phenotype of mice with the 126 bp (ΔCR2) deletion near TSS2, animals homozygous for the Δ50, mut5, and mut3 alleles were all born in normal Mendelian proportion, lived into adulthood, were fertile, and appeared to be healthy.

### The Δ50 and mut5 alleles impair GB formation

Examining the anatomy of the HPB system in adult mice homozygous for these new alleles revealed that *Sox17^Δ50/Δ50^* mice lacked both a GB and CD (Fig. 1B), *Sox17^mut5/mut5^* mice had an intermediate phenotype of GB hypoplasia (Fig. 1C). *Sox17^mut3/mut3^* mice exhibited no apparent differences in GB size or cellular morphology when compared to *Sox17^+/+^* animals (Fig. 1D; Fig. S1E). Inspection of animals heterozygous for the *Sox17^Δ50^* or *Sox17^mut5^* alleles revealed intermediate phenotypes in which the GB size was less severely affected compared to homozygous mutants (Fig. 1B-D). The finding that GB size varied depending on the specific allele and its zygosity suggested a *Sox17* gene dosage effect. Other internal organs such as liver, pancreas, spleen, stomach, intestine, kidney, lung, heart, testis, and ovary of *Sox17^Δ50/Δ50^* mice had no apparent abnormalities in size, color or general structure. Hematoxylin and eosin (H&E) staining of their pancreas, liver and testis showed no appreciable differences in cellular morphology or tissue structure (Fig. S1A-C). While RT-qPCR analysis of whole embryo mRNA from E9.5 *Sox17^Δ50/Δ50^* embryos revealed a reproducible but small increase in the liver hallmark gene *Hnf4a* (Fig. 1E), analysis of additional well-established transcriptional markers for lung, esophagus, intestinal tract, pancreas, ductal system, arterial/venous/lymphatic endothelium and hematopoietic tissue revealed no differences compared to controls (Fig. 1E; Fig. S1D), suggesting that the phenotype is limited to the GB and EHBD system.

### GB and EHBD formation is *Sox17* dependent

**Fig. 2.**
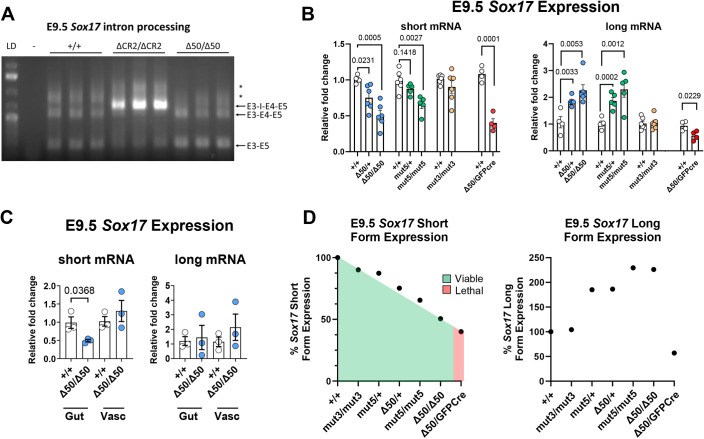
**Mutations within the *Sox17* TSS2 promoter differentially alter *Sox17* expression.** (A) RT-PCR assessment of intron (I) processing between exon (E) 3 and 4 in *Sox17* long mRNA forms in *Sox17^+/+^*, *Sox17^ΔCR2/ΔCR2^* and *Sox17^Δ50/Δ50^* embryos at E9.5. *n*=3. LD, ladder. (B) RT-qPCR analysis of *Sox17* short and long mRNA forms from E9.5 (without head and tail) embryos. Unpaired two-tailed Student's *t*-test. *Sox17* Short Form: *Sox17^+/+^*, *n*=4; *Sox17*^Δ*50/+*^*, n*=6, *P*=0.0231; *Sox17*^Δ*50/*Δ*50*^, *n*=6; *P*=0.0005. *Sox17^+/+^*, *n*=6; *Sox17^mut5/+^*, *n*=5, *P*=0.1418; *Sox17^mut5/mut5^*, *n*=5; *P*=0.0027. *Sox17^+/+^*, *n*=6; *Sox17^mut3/mut3^*, *n*=6; *P*=0.2364. *Sox17^+/+^*, *n*=4; *Sox17*^Δ*50/GFPCre*^, *n*=4; *P*=0.0001. *Sox17* long form: *Sox17^+/+^*, *n*=4; *Sox17*^Δ*50/+*^, *n*=6, *P*=0.0033; *Sox17*^Δ*50/*Δ*50*^, *n*=6; *P*=0.0053. *Sox17^+/+^*, *n*=6; *Sox17^mut5/+^*, *n*=5, *P*=0.0002; *Sox17^mut5/mut5^*, *n*=6; *P*=0.0012. *Sox17^+/+^*, *n*=6; *Sox17^mut3/mut3^*, *n*=6; *P*=0.8776. *Sox17^+/+^*, *n*=4; *Sox17*^Δ*50/GFPCre*^, *n*=4; *P*=0.0229. (C) RT-qPCR analysis of *Sox17* long and short mRNA forms in *Sox17^+/+^* and *Sox17*^Δ*50/*Δ*50*^ sorted fore-/mid-gut endoderm (EPCAM^+^/CXCR4^+^) and vascular endothelial cells (CD31^+^) at E9.5. Unpaired two-tailed Student's *t*-test. *Sox17* short form in the gut, *n*=3, *P*=0.0368. *Sox17* short form in the vasculature, *n*=3, *P*=0.4235. *Sox17* long form in the gut, *n*=3, *P*=0.8004. *Sox17* long form in the vasculature, *n*=3, *P*=0.3584. (D) Plotted fold changes of Sox17 long and short mRNA expression across the allelic series. Plotted fold changes were calculated by averaging fold change measurements from each condition analyzed in Fig. 2B. Data are mean±s.e.m.

Previously, we reported that the ΔCR2 deletion allele impaired RNA splicing of TSS1-derived long-form *Sox17* mRNAs (Trinh et al., 2022). In contrast, PCR analysis of long-form mRNA in *Sox17^Δ50/Δ50^* embryos showed no intron retention (Fig. 2A). To quantitatively assess the effects of specific CREs in the TSS2 promoter on *Sox17* expression levels, we used primers in an RT-qPCR assay that were specific for the long or short *Sox17* mRNAs on RNA isolated from the mid-regions (head and tail removed) at E9.5 from embryos in this allelic series. In addition, to assess how HPB development was affected by an even further reduction of *Sox17* expression, we crossed the Δ50 allele to a *Sox17* null allele (*Sox17^GFPCre^*) to generate compound heterozygous animals. The resulting *Sox17*^Δ*50/GFPCre*^ animals displayed perinatal lethality due to BA, as described below. Analysis by RT-qPCR showed no significant change in *Sox17* short mRNA levels in *Sox17^mut3/mut3^* embryos*,* whereas *Sox17^mut5/mut5^* embryos displayed ∼35% reduction, *Sox17*^Δ*50/*Δ*50*^ embryos ∼50% reduction, and *Sox17*^Δ*50/GFPCre*^ embryos ∼60% reduction (Fig. 2B). Additionally, *Sox17*^Δ*50/+*^ embryos had ∼25% reduction and *Sox17^mut5/+^* embryos only a ∼13% reduction (Fig. 2B). Expression of the long form of *Sox17* RNA was also determined in the same samples but exhibited no clear pattern of alteration (Fig. 2B).

RT-qPCR analysis of flow-sorted cells from posterior foregut and midgut endoderm (EPCAM^+^ CXCR4^+^) and/or vascular endothelial cells (CD31^+^) collected at E9.5 confirmed that the reduction of short *Sox17* mRNA in *Sox17*^Δ*50/*Δ*50*^ embryos was endoderm-specific (Fig. 2C). Similarly, levels of long-form *Sox17* mRNA trended upwards in separated gut and vasculature cell populations, but not to statistical significance (Fig. 2C). Plotting RNA-level averages against a systematic stepwise phenotype-severity scale, reflecting alterations in both GB and EHBD development and animal survival, produced a negative linear dependency, suggesting that reducing *Sox17* short-form mRNA levels below ∼50% is lethal (Fig. 2D). In contrast to the nearly linear association in expression of short-form RNA, the long-form *Sox17* mRNA exhibited a highly non-linear profile (Fig. 2D).

### Effects of reduced *Sox17* levels on HPB bud outgrowth and transcriptome characteristics in *Sox17^Δ50/Δ50^* mice

**Fig. 3.**
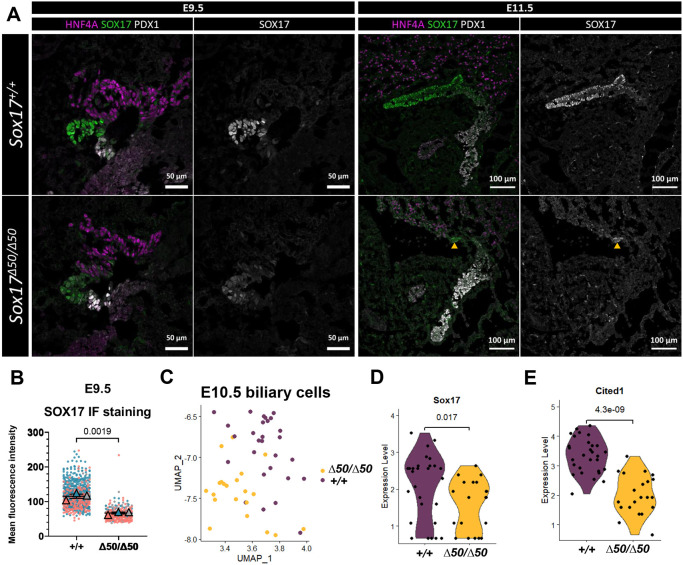
**Endoderm-specific reduction of *Sox17* short mRNA leads to failure in elongation of biliary bud.** (A) Immunofluorescent staining of HPB markers (SOX17 in green, biliary bud; HNF4A in magenta, liver bud; PDX1 in white, pancreatic bud) at E9.5 and E11.5 of WT and *Sox17^Δ50/Δ50^* embryos. Arrowheads indicate remnant of the biliary bud that fails to elongate. (B) Quantification of SOX17 immunolabeling signal in E9.5 WT and *Sox17^Δ50/Δ50^* embryos. Each dot represents a single cell, the different colors represent data points from different embryos, and the colored triangle-shaped points are average measurements from each different embryo. Unpaired two-tailed Student's *t*-test, *n*=3, *P*=0.0019. Data are mean±s.e.m. (C) UMAP of recovered biliary cells from E10.5 WT and *Sox17^Δ50/Δ50^* embryos. Colors indicate different genotypes based on hashtag barcodes. (D) Violin plot of *Sox17* expression in recovered biliary cells. Each dot represents a single cell. Unpaired two-tailed Student's *t*-test, *P*=0.017. (E) Violin plot of *Cited1* expression in recovered biliary cells. Each dot represents a single cell. Unpaired two-tailed Student's *t*-test, *P*=4.3e-09.

To determine when and how the ∼50% reduction in *Sox17* expression in *Sox17^Δ50/Δ50^* mice impairs GB and CD development, we performed immuno-labeling studies of the HPB buds at E9.5 and E11.5, two representative timepoints in the segregation and outgrowth of hepatic, pancreatic and biliary cell fates. In marked contrast to the profound impairment of HPB development in *Sox17^ΔCR2/ΔCR2^* embryos, in which a few remaining pancreatic progenitor cells were intermingled with liver and biliary progenitor cells (Trinh et al., 2022), HPB progenitor cells in *Sox17^Δ50/Δ50^* embryos successfully segregated into three distinct lineages of liver, pancreas, and biliary cells at E9.5 as marked by HNF4A, PDX1, and SOX17, respectively (Fig. 3A). However, at E11.5, 2 days later, the nascent biliary bud in *Sox17^Δ50/Δ50^* embryos failed to elongate (Fig. 3A; Fig. S2). At these stages, no obvious differences in proliferation or apoptosis were observed in HPB buds compared to controls (Fig. S3). Indirect quantitation of SOX17 using immunofluorescence staining and pixel-intensity measurements revealed a 42% reduction of staining signal, consistent with a reduction in SOX17 protein within E9.5 biliary progenitors (Fig. 3B) that roughly corresponds to the ∼50% reduction in short-form *Sox17* mRNA as determined by RT-qPCR. These results indicate that reduction of short-form *Sox17* expression from TSS2 impedes development of the nascent biliary cells, impairing or preventing formation of the GB and CD.

To better understand how the reduced *Sox17* expression in the *Sox17^Δ50/Δ50^* mice affects GB and EHBD development, we next performed single-cell RNA-sequencing (scRNA-seq) of EPCAM^+^ flow-sorted epithelial cells from the posterior foregut and midgut region of micro-dissected E10.5 embryos. This analysis identified nine distinct cell types – foregut, midgut, lung, liver, biliary, pancreas, pancreatic islet, blood and neural cells (Fig. S4A,B) – of which 18 and 28 cells were of biliary origin from *Sox17^Δ50/Δ50^* and *Sox17*^+/+^ mice, respectively (Fig. 3C). Despite the low number of biliary progenitor cells, differential expression analysis using MAST (model-based analysis of single-cell transcriptomics) revealed a total of ten dysregulated genes (Fig. S4C). The most clearly impaired genes in the *Sox17^Δ50/Δ50^* embryos were *Sox17*, which validates our previous results, and *Cited1* (CBP/p300-interacting transactivator with Glu/Asp-rich carboxy-terminal domain 1) (Fig. 3D,E). A reduction in *Cited1* expression was previously observed in GBs from *Sox17^+/−^* mice at E15.5 (Higashiyama et al., 2017). To determine whether the ∼50% reduction of *Sox17* expression in *Sox17^Δ50/Δ50^* mice might also affect liver development, we performed bulk RNA-seq of E12.5 embryonic livers. While some minor changes in a few genes (Fig. S4D,F) were observed, taken as a whole, our results indicate that the *Sox17* reduction in *Sox17^Δ50/Δ50^* embryos, while greatly impairing GB and EHBD development, has very little effect on the liver itself.

### Transcriptional profiling of *Sox17^mut5/mut5^* GBs

**Fig. 4.**
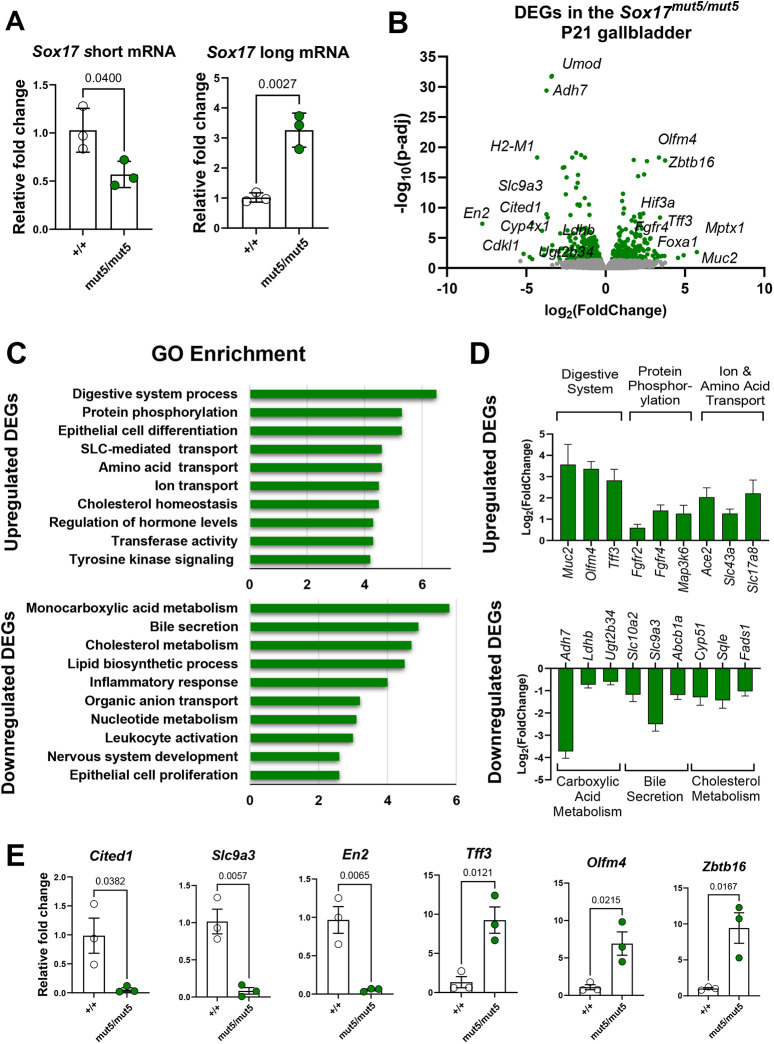
**Transcriptional profile of *Sox17^mut5/mut5^* GBs.** (A) RT-qPCR analysis of *Sox17* RNA forms in the *Sox17^mut5/mut5^* P21 gallbladder (GB). Unpaired two-tailed Student's *t*-test. *Sox17* short form, *n*=3, *P*=0.0400. *Sox17* long form, *n*=3, *P*=0.0027. (B) RNA-seq analysis. Volcano plot [Log_2_ FoldChange (FC) over −log_10_(*P*-adj)] showing differentially expressed genes (DEGs) with green dots in *Sox17^mut5/mut5^* versus *Sox17^+/+^*. (C) Gene Ontology (GO) Term enrichment analysis of up- and downregulated genes. Selected top enriched terms are shown, with the full list in Table S1. (D) Differential expression of representative genes within enriched GO terms shown in C. Text indicates functional associations. (E) RT-qPCR validation of select up- and downregulated genes in P21 GBs from *Sox17^mut5/mut5^* and WT mice. Unpaired two-tailed Student's *t*-test. All genes measured have an *n*=3. *Cited1*, *P*=0.0382. *Slc9a2*, *P*=0.0057. *En2*, *P*=0.0065. *Tff3*, *P*=0.0121. *Olfm4*, *P*=0.0215. *Zbtb16*, *P*=0.0167. Data are mean±s.e.m.

Due to the very low number of cells recoverable from the small GB primordium, limiting our ability to identify additional differentially-expressed genes at that timepoint, we also performed bulk RNA-seq on the hypomorphic GBs present in *Sox17^mut5/mut5^* mice. At postnatal day (P) 21, *Sox17^mut5/mut5^* GBs exhibited a downregulation in short-form *Sox17* mRNA and increased long-form mRNA (Fig. 4A), similar to that in the mid-region of E9.5 embryos (Fig. 2B). Bulk RNA-seq of GB RNA comparing *Sox17^mut5/mut5^* and *Sox17^+/+^* by differential expression analysis revealed 221 upregulated genes and 258 downregulated genes (using the *P*_adj_<0.05 threshold; Fig. 4B), which again included *Cited1*. Gene ontology (GO) term enrichment analysis revealed that upregulated genes were linked to digestive system process, epithelial cell differentiation, protein phosphorylation and amino-acid transport, and that downregulated genes were enriched for monocarboxylic acid metabolism, bile secretion, and cholesterol metabolism (Fig. 4C). Complete differential expression and GO term enrichment analysis can be found in Table S1.

Conspicuously, among the top upregulated genes were *Tff3*, *Muc2*, *Agr2*, and *Spink4*, markers of intestinal goblet cells (Kashiwagi et al., 2001; Aihara et al., 2017; Hensel et al., 2024), *Olfm4*, a marker for intestinal progenitor cells (van der Flier et al., 2009), and *Fgfr4*, a receptor that inhibits bile synthesis in the liver (Chiang, 2009) (Fig. 4D). At the same time, genes involved in bile-acid metabolism (such as *Adh7*, *Ldhb*, and *Ugt2b34*) (Schueren et al., 2014; Feng et al., 2021; Liu et al., 2023), bile secretion (*Slc10a2*, *Slc9a3*, *Abcb1a*) (Chiang, 2009; Xu et al., 2018; Pizzagalli et al., 2021), and cholesterol metabolism (such as *Cyp51*, *Sqle*, and *Fads1*) (Rozman, 2000; Yuan et al., 2005; Keber et al., 2011; Gromovsky et al., 2018; Heravi et al., 2022; Huang et al., 2022; You et al., 2022) were downregulated (Fig. 4D). These results indicate that a modest reduction of *Sox17* expression in *Sox17^mut5/mut5^* mice not only causes decreased GB size but also impaired GB epithelial cell identity. Moreover, since a principal GB function is to store, concentrate and regulate bile-acid composition (Housset et al., 2016), the observed decrease in genes related to these processes, and increase in intestinal epithelial markers, suggests potential functional abnormalities. We validated the expression changes of selected *Sox17*-dependent genes using RT-qPCR, confirming the increased *Tff3*, *Olfm4* and *Zbtb16*, and decreased *Cited1*, *En2* and *Slc9a3* expression (Fig. 4E). Since *Zbtb16*, *Cited1* and *En2* are transcriptional regulators, they may be core components of a SOX17-dependent gene-regulatory network in GB epithelial cells.

### Marked abnormality of GBs from *Sox17^mut5/mut5^* mice

**Fig. 5.**
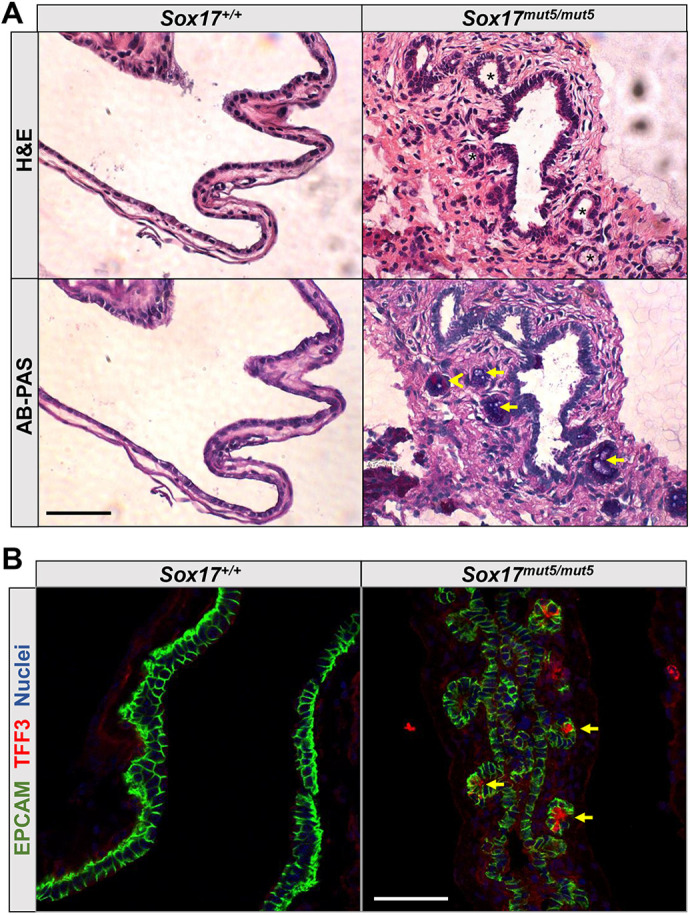
***Sox17^mut5/mut5^* GB epithelium displays metaplastic changes.** (A) Hematoxylin and eosin (H&E) and AB-PAS staining of WT *Sox17^+/+^* and *Sox17^mut5/mut5^* GBs. Asterisks indicate intramural gland-like and crypt-like structures within *Sox17^mut5/mut5^* GB epithelium. The structures contain gastric pyloric-type gland cells producing neutral mucins stained red (arrowhead) and intestinal goblet-like cells producing acidic mucins stained blue (arrows). (B) Immunofluorescent staining of *Sox17^+/+^* and *Sox17^mut5/mut5^* GBs with antibodies against epithelial cell marker EPCAM (green) and intestinal goblet cell marker TFF3 (red), with nuclei stained with TO-PRO3 (blue). Arrows indicate TFF3^+^ goblet-like cells within *Sox17^mut5/mut5^* GB epithelium. Scale bars: 50 μm.

To determine whether the gene-expression changes in *Sox17^mut5/mut5^* mice could be explained as a form of intestinal or gastric metaplasia, a phenotype previously noted in the GBs of human cholecystectomy patients (Albores-Saavedra et al., 1986; Duarte et al., 1993; Fukumura et al., 2022), we examined the histology of P21 GBs using H&E and immunofluorescence staining (Fig. 5). H&E and Alcian Blue/Periodic Acid-Schiff (AB-PAS) stain showed that *Sox17^mut5/mut5^* GB epithelium displayed features characteristic of metaplastic transformation, with visible intramural gastric gland-like or intestinal crypt-like structures that are positive for neutral gastric and acidic intestinal mucins and that contain pyloric gland-like and intestinal goblet-like cells (Fig. 5A,B). Co-immunostaining with epithelial cell-surface marker EPCAM and intestinal goblet-cell marker TFF3 confirmed the presence of goblet-like TFF3^+^ cells located in ‘crypt-like structures’ of *Sox17^mut5/mut5^* GB epithelium (Fig. 5B). Conversely, wild-type (WT) GB epithelial cells were characterized by a strong EPCAM staining and single cell layer organization. In addition, we surveyed markers of EHBD peribiliary glands (such as *Axin2*, *Muc6*, *Ccnb2*, *Lgr6*) (Rimland et al., 2021; Singh et al., 2023) within our *Sox17^mut5/mut5^* GB RNA-seq dataset and found no significant differences in gene expression. These results indicate that a decrease in *Sox17* expression causes alterations in the GB epithelium similar to the gastric and intestinal metaplasia associated with GB cancer in humans.

### Effects on GB and EHBD development of critical reductions in *Sox17* expression

**Fig. 6.**
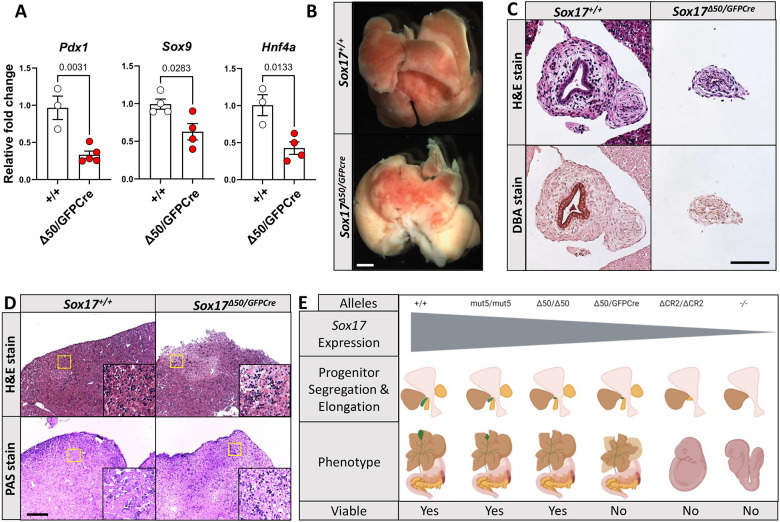
**The *Sox17*^Δ*50/GFPcre*^ compound heterozygous genotype is lethal due to biliary atresia.** (A) RT-qPCR analysis of *Sox17*^Δ*50/GFPCre*^ embryos (without head and tail) for HPB markers: *Pdx1*, pancreas and duodenum; *Sox9*, ductal system; *Hnf4a*, liver. Unpaired two-tailed Student's *t*-test. Data are mean±s.e.m. *Pdx1*: *Sox17^+/+^*, *n*=3; *Sox17*^Δ*50/GFPCre*^, *n*=5; *P*=0.0031. *Sox9*: *Sox17^+/+^*, *n*=4; *Sox17*^Δ*50/GFPCre*^, *n*=4; *P*=0.0283. *Hnf4a*: *Sox17^+/+^*, *n*=3; *Sox17*^Δ*50/GFPCre*^, *n*=4; *P*=0.0133. (B) Representative stereoscope images of P1 livers. (C) Representative hematoxylin and eosin (H&E) and ductal marker DBA staining of extrahepatic biliary ducts within the P1 livers. (D) Representative H&E (top) and periodic acid Shiff (PAS) (bottom) staining of the edge of the P1 livers shows areas containing hepatocytes devoid of glycogen granules (purple in PAS stain) and increased infiltration with immune cells in *Sox17*^Δ*50/GFPCre*^ livers. Insets show magnification of boxed areas. (E) Model of *Sox17* dosage-dependent development in the HPB system. WT embryos are viable with normal size gallbladders (GBs). Mut5 mutant embryos are viable with ∼35% reduction of endodermal *Sox17* expression and have a hypoplastic GB. *Sox17*^Δ*50/*Δ*50*^ embryos are viable with ∼50% reduction of endoderm-specific *Sox17* and fail to develop a GB or cystic duct (CD). *Sox17*^Δ*50/GFPCre*^ embryos have a ∼60% reduction of *Sox17* and die perinatally due to biliary atresia. CR2-null embryos with incomplete loss of *Sox17* die due to a failure to segregate cells in the foregut diverticulum. *Sox17*-null embryos are lethal with failure in axis rotation and lack *Pdx1* expression. Created in BioRender. Finnel, R. (2025) https://BioRender.com/e79s990. Scale bars: 2.5 mm (B); 200 μm (C); 100 μm (D).

We next characterized the effect of the ∼60% reduction of *Sox17* expression in compound heterozygous (*Sox17*^Δ*50/GFPCre*^) mice. We first observed that *Sox17*^Δ*50/GFPCre*^ animals, unlike *Sox17*^Δ*50/*Δ*50*^ mice, died within the first few days after birth, with none remaining alive at weaning (*P*=0.000063, Chi-squared analysis). Analysis of several HPB system markers in E9.5 *Sox17*^Δ*50/GFPCre*^ embryos showed reduced *Pdx1* (pancreas and duodenum), *Sox9* (ductal), and *Hnf4a* (liver) expression (Fig. 6A), with the latter being in marked contrast to the increased *Hnf4a* seen in the *Sox17*^Δ*50/*Δ*50*^ embryos of the same age (Fig. 1E). The gross morphology and histology of livers from *Sox17*^Δ*50/GFPCre*^ mice at P1, about a day before expected death, revealed signs of cholestasis, with increased blanching of the distal hepatic lobes due to necrosis, disruption of the hepatic capsule, and increased inflammation (Fig. 6B,D), features reminiscent of *Sox17* heterozygous-null mice in a C57BL/6 background (Uemura et al., 2013). Consistent with biliary obstruction being the principal cause of death, H&E and DBA staining of *Sox17*^Δ*50/GFPcre*^ tissue showed the total absence of the GB, a markedly underdeveloped ductal epithelium, and a non-patent biliary duct (Fig. 6C). These results indicate that only a small additional reduction in *Sox17* expression, from ∼50% to ∼60%, is enough to impair GB and EHBD development below a threshold where BA and cholestasis-induced hepatitis occur, causing perinatal lethality (Fig. 6E). Similar to the *Sox17*^Δ50/Δ50^ animals, earlier-stage liver development in E12.5 *Sox17*^Δ*50/GFPCre*^ mice showed effects on only a few genes by bulk RNA-seq (Fig. S4E,G).

### SOX17 binds the m5 region in the TSS2 promoter-proximal region

**Fig. 7.**
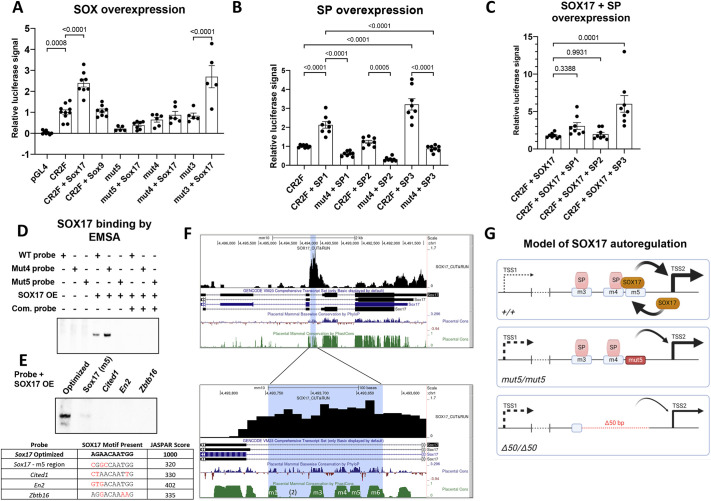
**SOX17^+^ autoregulation and potential interactions with SP/KLF factors enhance *Sox17* expression to levels necessary for GB formation.** (A) *Luciferase* reporter assay testing WT (CR2F) and mutant (mut3, mut4, mut5) TSS2 promoter activity in mouse differentiated definitive endoderm with overexpression of SOX17 (*n*=8, *P*<0.0001) or SOX9 (*n*=8, *P*>0.9999) while using the pGL4 empty vector as a negative control (Mut3 with Sox17 overexpression, *n*=5, *P*<0.0001). Each dot represents one well of endoderm across at least three differentiation experiments. One-way ANOVA. (B) *Luciferase* reporter assay testing WT (CR2F) and mutant (mut4) TSS2 promoter activity in mouse differentiated definitive endoderm with overexpression of SP1 (*P*<0.0001), SP2 (*P*=0.8969) and SP3 (*P*<0.0001). *n*=8. One-way ANOVA. Each dot represents one well of endoderm across at least three differentiation experiments. (C) *Luciferase* reporter assay testing WT (CR2F) and mutant (mut4) TSS2 promoter activity in mouse differentiated definitive endoderm with overexpression of SOX17+SP1 (*P*=0.3388), SOX17+SP2 (*P*=0.9931) and SOX17+SP3 (*P*=0.0001). Each dot represents one well of endoderm across at least three differentiation experiments. *n*=8. One-way ANOVA. (D) Electrophoretic mobility shift assay (EMSA) demonstrates direct binding of SOX17 at m5 region within TSS2 promoter. The binding of SOX17 to WT and the mut4 mutant but not to the mut5 mutant probe generates shifted bands. SOX17 OE, *Sox17* overexpression lysate from 293T cells; Com. probe, competing probe. Full image is in Fig. S5. (E) EMSA comparing the binding of Sox17 at the m5 region in TSS2 and several binding sites that lie near genes that are differentially regulated in the GBs of *Sox17^mut5/mut5^* mice. While strong binding occurs on the optimized SOX17 binding motif, much weaker or no binding occurs at all other sites under the conditions of this experiment. Full EMSA image is in Fig. S5. Bases marked in red deviate from the SOX17 consensus motif. (F) Within the *Sox17* TSS2 promoter (blue highlight), SOX17 CUT&RUN data shows binding of SOX17 within *Sox17* in the cXEN extra-embryonic endoderm stem cell line (GEO series GSE213661, sample GSM6591690; Pham et al., 2023). Top panel viewing window, GRCm38/mm10: chr1:4,490,931-4,496,413. Bottom panel viewing window, GRCm38/mm10: chr1:4,493,565-4,493,823. (G) Model of the SOX17-SP interaction at CR2 which regulates TSS2 via a *Sox17*^+^ autoregulatory loop. In +/+ animals, SOX17 binds to m5 within the TSS2 promoter to positively regulate *Sox17* expression. This interaction is also dependent on binding of an SP factor at m4. In *Sox17^mut5/mut5^* animals, the autoregulatory loop is broken as the SOX17 binding site is mutated, leading to a ∼35% reduction in *Sox17* expression and a hypoplastic gallbladder (GB). Lastly, in the Δ50 bp deletion, three CREs (m3, m4 and m5) are disrupted. This deletion does not allow for SOX17 binding, thereby disrupting the positive autoregulatory loop. In addition, TF binding at m3 and m4 is also disrupted, leading to a ∼50% reduction in *Sox17* expression and lack of GB organogenesis. Created in BioRender. Finnel, R. (2025) https://BioRender.com/e79s990. Data are mean±s.e.m.

To better understand how the m3-m4-m5 region and the m3 and m5 CREs regulate TSS2-based *Sox17* expression, and to clarify the identity of the TFs that bind within this region, we analyzed the effects of protein overexpression on a series of CR2-driven *Luciferase* (*Luc*) reporter plasmids containing specific CRE mutations in mESC-derived endoderm. Since the m5 CRE was predicted to bind a SOX protein, we sought to determine whether the CR2-*Luc* reporter gene could be activated by SOX17 or SOX9, both of which are strongly expressed within the developing ductal lineage of the HPB system. SOX17 but not SOX9 increased reporter-gene expression and the enhancing effect of SOX17 was diminished when the m5 but not m3 CRE was mutated, confirming the specific activation by SOX17 via the m5 CRE (Fig. 7A). In addition to the m5 CRE being necessary for *Sox17* activation, the neighboring m4 region is also required. When the latter was mutated, the ability of SOX17 to enhance reporter gene expression was eliminated, indicating that a molecular interaction between factors binding the m4 and m5 CREs likely also contributes to *Sox17* expression from TSS2.

Since the m4 CRE motif is GC-rich, with the potential to bind various SP/KLF factors, we compared the effects of SP1, SP2 and SP3 overexpression, together with SOX17, on reporter-gene activity. All three SP factors caused increased reporter expression, and again in an m4-dependent manner (Fig. 7B). The greatest increase was with SOX17 overexpressed with SP3 (Fig. 7C). We also performed gel electrophoretic mobility-shift assay (EMSA) to further demonstrate that the m5 CRE can be bound by SOX17, as indicated by the band shift with WT but not with the mut5 oligonucleotide probe (Fig. 7D; Fig. S5).

Finally, to directly assess the relative binding affinities of the SOX17 binding site in the m5 region and that of several other SOX17 binding sites located near genes that are differentially regulated in the GBs from *Sox17^mut5/mut5^* mice, we performed an additional EMSA experiment that compared binding of these sites to an optimal SOX17 motif (Fig. 7E). While the optimal site showed strong binding, all other sites, each of which had lower JASPAR scores, showed little or no binding under the conditions of the experiment. These results indicate that the SOX17 binding site located in the TSS2 promoter and sites near other differentially regulated genes are suboptimal, and therefore are likely to bind SOX17 with a lower affinity. Additionally, inspection of publicly available ChIP-seq data (Pham et al., 2023) revealed that SOX17 binds to the CR2 region in extra-embryonic endoderm (Fig. 7F). Additionally, ChIP-seq data for SOX17 from human iPSCs differentiated to definitive endoderm (Mukherjee et al., 2022) indicates the existence of SOX17 binding peaks, which localize over SOX17 motifs in the vicinity of the SOX17-dependent genes identified in E10.5 GB progenitors and P21 GB tissue from mice (Fig. S6). Consistent with the notion that high SOX17 levels, or in cooperation with a partner protein, are necessary for activation of SOX17 response elements, many of the ChIP-seq SOX17 sites identified in human endoderm also deviate from the consensus SOX17 binding sequence (Fig. S6E,F). Together, the exquisite sensitivity of the developing GB and EHBD system in mice to different levels of *Sox17*, the presence of a suboptimal SOX17 binding site in TSS2 promoter, and our findings using reporter gene assays indicate the existence of a feed-forward, positive autoregulatory loop that enhances *Sox17* expression during HPB development (Fig. 7G).

## DISCUSSION

### Development of the GB and EHBD system is dependent on *Sox17*

These studies clearly demonstrate the exquisite sensitivity of the developing GB and EHBD system to expression levels of *Sox17*. A mere ∼25% reduction of the short *Sox17* mRNA, as occurs in *Sox17*^Δ*50/+*^ mice, causes a reduction in GB size, whereas *Sox17*^Δ*50/*Δ*50*^ mice, with ∼50% reduction, totally lack a GB and CD. Given that the size of the GB was visibly reduced in *Sox17^mut5/+^* mice, an even smaller reduction of *Sox17* expression likely also has an effect (Fig. 1C). When *Sox17* expression falls below ∼50%, as occurs in *Sox17*^Δ*50/GFPCre*^ mice, development of the GB and EHBD becomes so impaired that the animals die of BA-induced cholestasis and subsequent hepatic necrosis. These findings not only indicate that there is a critical threshold of *Sox17* expression necessary for GB and EHBD development, they also suggest region- and/or developmental event-specific sensitivities of the nascent GB and EHBD system to different SOX17 concentrations.

Our findings are consistent with the gene dosage effects reported for both SOX9 (Naqvi et al., 2023) and SOX2 (Abatti et al., 2023) and point to crucial binding affinity differences among SOX CREs. Using the terminology of Naqvi et al., we speculate that SOX17 binding sites essential for formation of the GB and EHBD system are ‘sensitive’ elements that require higher SOX17 levels for binding, whereas SOX17 binding sites in early endoderm and initial HPB segregation are ‘buffered’ elements that require lower amounts of SOX17 for binding. Recently it has been reported that suboptimal TF binding sites may be essential for tissue development (Farley et al., 2015; Jindal et al., 2023; Lim et al., 2024). In the case of the GB and EHBD system, low-affinity sites for SOX17 may determine key features of GB and EHBD formation through their exquisite sensitivity to concentrations of SOX17.

### Formation of the GB and EHBD system requires a positive *Sox17* autoregulatory loop

Our studies also indicate that specific CREs in the TSS2 promoter of *Sox17* are crucial for GB and EHBD system development. Both the Δ50 bp and smaller mut5 transversion mutation cause reduced levels of the endoderm-enriched *Sox17* short-form mRNA and SOX17 protein in biliary progenitor cells. The TSS2 promoter region likely binds SP or KLF factors (at m1, m3 and m4) and SOX17 (at m5). The close proximity of binding sites for these factors, the known ability of SOX family members (e.g. SOX6, SOX8, SOX9, SOX10 and SRY) to interact with SP1 and SP3 (Melnikova et al., 2000a,b; Wissmuller et al., 2006; Yokoyama et al., 2006; Renard et al., 2012), and the requirement of the m4 CRE for SOX17 to transactivate the TSS2 promoter in reporter-gene assays in cultured endodermal cells, suggest that yet-to-be-defined molecular interactions between SOX17 and SP/KLF factors are required for SOX17 to regulate TSS2 promoter activity.

The existence of a positive autoregulatory loop operating at the *Sox17* TSS2-associated promoter region provides a plausible mechanism for driving SOX17 to sufficiently high levels necessary for mediating SOX17-based activation at low-affinity SOX17 binding sites within target genes involved in development of the GB and EHBD system. Indeed, a positive autoregulatory mechanism involving *Sox17* was previously suggested by a study involving *Xenopus* (Howard et al., 2007). Moreover, the high similarity of the human SOX17 promoter with the murine TSS2 promoter studied here suggests the use of this regulatory mechanism in humans (Trinh et al., 2022). Together with the results of this study, we suggest that lower levels of SOX17 are sufficient to enable the segregation of biliary, pancreatic, and hepatic progenitor cells during HPB development, but high SOX17 levels are required to efficiently form the GB and EHBD. Indeed, based on our EMSA analysis, the m5 CRE in the TSS2 promoter is a low-affinity or ‘sensitive’ SOX17 response element that requires high levels of SOX17 for binding. In addition, our fusion gene studies indicate the need for SOX17 to interact with one or more SP/KLF factors at the m4 motif, as illustrated in the model shown in Fig. 7G. Indeed, considering that the Δ50 mutation obliterates the m3, m4 and m5 motifs, and that the transversion mutation of m3 has little effect, it can be inferred that the more pronounced phenotype of the Δ50 bp mutation compared to the transversion mutation of m5 is likely due to the m4-m5 interaction.

### An undefined compensatory response by the TSS1 promoter region

The upregulation of the TSS1-derived *Sox17* long-form mRNA in *Sox17^Δ50/Δ50^*, *Sox17*^Δ*50/+*^, *Sox17^mut5/mut5^* and *Sox17^mut5/+^* suggests a compensatory mechanism that responds to the decreased TSS2-derived transcription of *Sox17* short-form mRNAs. However, previous studies have shown that TSS2 is active predominantly in endoderm with very low activity in vascular endothelium, while TSS1 is only active in vascular endothelium (Liao et al., 2009; Trinh et al., 2022). However, flow-sorted endoderm and vascular endothelial cells from E9.5 *Sox17*^Δ*50/*Δ*50*^ suggest, but do not show a significant increase in the expression of the long form of *Sox17* mRNA in both lineages (Fig. 2C). Additional experimentation is required to determine both the mechanism and significance of these differences.

### A decrease in *Sox17* expression adversely affects adult GB epithelium

A modest decrease in *Sox17* levels in *Sox17^mut5/mut5^* animals leads not only to hypoplastic GBs but also to alterations in GB transcriptome and tissue morphology. The observed increase in expression of intestinal-cell markers and decrease in bile-acid transport and metabolism genes indicate that proper *Sox17* levels are necessary for maintenance of epithelial cell identity and function in adult GBs. *Sox17^mut5/mut5^* GBs showed features consistent with gastric and intestinal metaplasia, a condition often observed in cholecystectomy tissues from patients with small gallstones and chronic cholecystitis, which is a possible step towards malignant carcinoma (Duarte et al., 1993; Mathur et al., 2012; Seretis et al., 2014; Kocaoz and Turan, 2019). Therefore, we speculate that gallstones or other physical obstructions that cause inflammation may cause dysplasia and/or metaplastic transformation by altering epithelial *Sox17* levels. In support of this idea, *SOX17* regulates cholangiocyte differentiation and functions as a tumor suppressor in cholangiocarcinoma (Merino-Azpitarte et al., 2017). Furthermore, in addition to antagonizing the pro-tumorigenic Wnt/β-catenin signaling pathway (Jia et al., 2010), SOX17 functions as a tumor suppressor for several types of cancer in other endoderm-derived organs (Du et al., 2009; Kuo et al., 2014; Li et al., 2015).

### Species-specific differences in the TSS2 promoter region do not explain the evolutionary loss of GBs

The GB has repeatedly undergone evolutionary loss in vertebrates, as has occurred repeatedly in rodents (Higashiyama et al., 2018). Since mutations in the TSS2 promoter region can attenuate or eliminate formation of the GB and CD in mice, we wondered whether sequence alterations in the TSS2 promoter might connect to the absence of GB in certain mammalian species. However, sequence comparisons of two GB-containing and two GB-absent rodent species showed no consistent differences (Fig. S7), with perfect and near-perfect sequence conservation in m5 and m4 CREs, respectively. Therefore, evolutionary differences in distal enhancers that regulate *Sox17* may cause these species-specific differences.

### Concluding comments

Our results demonstrate the exquisite sensitivity of the developing GB and EHBD system to alterations in *Sox17* expression and suggest that specific developmental events may differ in their requirement for SOX17. In addition, we also identified a CRE within the TSS2 promoter region that, by its ability to bind SOX17, creates a positive autoregulatory loop that may boost *Sox17* gene expression to levels sufficient to activate SOX17-dependent response elements within GB and EHBD progenitor cells during hepato-pancreato-biliary system formation.

## MATERIALS AND METHODS

### Derivation of new strains

Three new mutant mouse alleles Δ50 (Rr271^em2Mgn^, MGI: 7461678), mut3 (Rr271^em3Mgn^, MGI: 7461680), and mut5 (Rr271^em4Mgn^, MGI: 7461681) were designed and produced by the Vanderbilt Genome Editing Resource in Nashville, TN, USA. The alleles were generated by pronuclear microinjection of a Cas9 ribonucleoprotein complex into fertilized zygotes from the mating of CD-1 mice. The injection solution contained 100 ng/μl WT SpCas9 protein (MilliporeSigma), a target-specific HPLC purified, chemically-modified sgRNA at 50 ng/μl (Synthego) and 50 ng/μl of a PAGE purified, 180 nt single stranded DNA (MilliporeSigma) in 10 mM Tris, 0.1 mM EDTA buffer at pH 7.6 (TEKnova, T0230). See Table S2 for crRNA and ssDNA sequences. Injected embryos were implanted into pseudo-pregnant CD-1 mice. Pups were weaned and tail biopsies were performed at 3 weeks of age. Founder mice carrying desired deletions were identified by PCR (see Table S3 for primer sequences) and confirmed by Sanger sequencing. Founder animals were backcrossed to WT CD-1 mice for three generations before interbreeding to produce homozygous mutant mice. These experimental procedures used were approved and monitored by the Vanderbilt Institutional Animal Care and Use Committee.

### Routine husbandry and genotyping

Mice were socially housed within Vanderbilt's animal facility with a 12 h light/12 h dark cycle. All mouse lines were maintained in an outbred Crl:CD-1 (ICR), or CD-1^®^ IGS background (Charles River, model #022) by breeding heterozygous males with WT CD-1 females obtained from Charles River. For genotyping, DNA was obtained from adult tail biopsy or embryonic yolk sac lysates. WT littermate embryos were used as controls for homozygous mutant embryos. Genomic DNA was extracted from adult tails or embryonic yolk sacs digested with 0.5 mg/ml Proteinase K (Thermo Fisher Scientific, EO0491) in lysis buffer (10 mM Tris pH 8.0, 100 mM NaCl, 10 mM EDTA and 0.5% SDS) at 55°C overnight.

### Cell lines

Contamination-free TL1 mESCs (Labosky et al., 1997) were cultured at 37°C in 5% CO_2_ on mitomycin C (Tocris, 3258/10)-treated DR4 mouse embryonic fibroblast (MEF) feeder cells using mESC complete medium prepared from Dulbecco's Modified Eagle Medium (DMEM) (Thermo Fisher Scientific, 11960-044) supplemented with 15% heat-inactivated fetal bovine serum (FBS) (R&D Systems, 11550), 1 mM non-essential amino acids (Thermo Fisher Scientific, 11140-050), 2 mM L-glutamine (Thermo Fisher Scientific, 25030-081), 1× gentamicin (Thermo Fisher Scientific, 15750-060), 103 U/ml mouse Leukemia Inhibitory Factor (mLIF) (MilliporeSigma, ESG1107) and 0.11 mM β-mercaptoethanol (Gibco, 21985-023) as previously described (Trinh et al., 2022). Cells were split 1:6-1:8 at ∼80% confluence using 0.25% trypsin (5 min at 37°C) (Thermo Fisher Scientific, 15050057). During differentiation, feeder MEFs were depleted from mESCs via trypsinization and the mixed cells were plated on gelatinized plates for 30 min to 1 h at 37°C, 5% CO_2_. After incubation, floating MEF-depleted mESCs were pelleted via centrifugation at 1000 rpm (200 ***g***) for 5 min, resuspended at desired concentrations, and then plated on gelatin-coated plates for further differentiation.

### Embryo collections

For stage-specific embryo collections, timed matings were performed by placing 8-week-old heterozygous male mice that had been single-housed with 1 to 2 group-housed heterozygous females that were similarly 6-8 weeks old. Detection of vaginal plugs were indicated as being 0.5 days post coitum (dpc) [or embryonic day (E) 0.5]. At the relevant developmental time points, embryonic tissues were micro-dissected in cold phosphate buffered saline (PBS) (Corning, 46-013-CM) and staged according to Theiler staging criteria.

### Sequence conservation analyses

Boundaries of mutated regions (Δ50: mm10 chr1:4,493,644-4,493,693; mut3: chr1:4,493,691-4,493,699; mut5: chr1:4,493,647-4,493,655) were determined based on sequence conservation analysis using the University of California, Santa Cruz (UCSC) genome browser (http://genome.ucsc.edu) on mouse assembly mm10 (conservation track using Multiz alignments and PHAST package) and VISTA-Point multiple genomes alignment tool (http://pipeline.lbl.gov/cgi-bin/gateway2) (Mayor et al., 2000) as defined previously (Trinh et al., 2022).

### Polymerase chain reaction

PCR reactions used the EconoTaq PLUS 2× Master Mix (Biosearch Technologies, 30035) standard protocol (see Table S3 for primer sequences) and the resulting products were analyzed using 2% agarose gel electrophoresis.

### RT-qPCR

RNA was extracted from embryos, GBs, sorted cells or cultured cells using Promega Maxwell^®^ 16 LEV simplyRNA Purification Kit (Promega, AS1280) then converted to cDNA using a High Capacity cDNA Reverse Transcription Kit (Thermo Fisher Scientific, 4368814) using manufacturer recommendations. cDNAs were then subjected to qPCR using SYBR™ Green PCR Master Mix (Thermo Fisher Scientific, 4309155) in conjunction with a Bio-Rad CFX Real time PCR instrument. The amplification program consisted of 95°C for 10 min followed by 45 cycles of 95°C for 15 s and 60°C for 1 min (see Table S4 for primer sequences). qPCR amplicons spanned exon junctions when possible and appeared as a single band at expected size when analyzed with agarose gel electrophoresis. Relative fold changes were calculated using double delta Ct method with *Gapdh* or *Hprt* as a housekeeping gene and WT samples as controls for normalization. Three technical replicates were obtained for each biological sample, which were then averaged before comparing to the appropriate control.

### GB analyses and measurement

GBs were dissected from 8- to 12-week-old animals, which were fasted overnight before dissection and placed into 1× PBS on ice. GBs were imaged on a Leica MZ 16 FA microscope. Images were analyzed using FIJI software (Schindelin et al., 2012), with the size being determined using the area of best-fit ellipse tool. For sectioning, GBs were either fixed in 10% formaldehyde overnight for paraffin sections or processed for immunofluorescent staining on frozen sections as described below.

### Liver analysis

Timed matings were performed and female mice were checked for pups daily at 19 to 21 dpc. Once pups were observed, they were collected for dissection at P0-P1. Livers were placed onto ice-cold PBS and imaged using a Leica MZ 16 FA fluorescent stereo microscope equipped with QImaging Retiga 4000R camera. Subsequently, livers were fixed in 10% formaldehyde for sectioning.

### DBA staining

Liver samples were fixed in a 10% formalin solution overnight, then placed in 70% ethanol and further processed at the Vanderbilt Translational Pathology Shared Resource (TPSR). Tissues were embedded in paraffin blocks and sectioned into 5 μm serial sections. For DBA staining, sections were first re-hydrated using xylene and rinsing in stepwise reductions of ethanol, followed by rinsing with tap water and PBS. Sections were then blocked with 3% bovine serum albumin (BSA) (MilliporeSigma, A3059) for 1 h at room temperature. Sections were incubated with DBA-biotin at 1:1000 in 3% BSA overnight at 4°C. Sections were then washed twice with PBST and once with PBS. Next, sections were incubated with 0.3% H_2_O_2_ in PBS for 15 min followed by a PBS wash. Using an ABC-HRP Kit (Vector Laboratories, PK-4000), sections were incubated with Avidin-HRP for 30 min at room temperature, followed by two PBS washes. Sections were incubated with a Vector DAB Kit (Vector Laboratories, SK-4100) before washing with tap water. Next, sections were counterstained with Eosin and then washed for 5 min with tap water. Sections were then dehydrated using a stepwise increase of ethanol before being treated with xylene. Slides were then sealed with Cytoseal and imaged using a Zeiss Axioplan 2 upright microscope equipped with a QImaging Retiga EXi camera.

### Histochemical staining

H&E staining and AB-PAS staining of paraffin-embedded tissue sections were performed by Vanderbilt TPSR using established protocols. Images were obtained using a Zeiss Axioplan 2 upright microscope equipped with a QImaging Retiga EXi camera.

### Immunofluorescence staining

E9.5-E11.5 embryos or P21 GBs were dissected in PBS then fixed in 2% paraformaldehyde for at least 1 h at room temperature before incubation in 30% sucrose in 1× PBS solution at 4°C overnight on a shaker. Intact embryos and tissues were then embedded in Tissue-Tek^®^ OCT compound (Sakura, 4583) on dry ice. Frozen embryos were cut into 8 μm thick sections using a Leica CM3050S cryostat, post-fixed with a cold acetone, and air-dried for 30 min followed by permeabilization with 0.3% Triton X-100 in PBS for 10 min at room temperature. Blocking was performed for 1 h at room temperature with 3% BSA in PBS before primary antibodies in the same solution were applied to samples for overnight incubation at 4°C (see Table S7 for an antibody list and a dilution factor). Tissue and embryo samples were washed three times (10 min each) with PBST (0.2% Tween in PBS) before being incubated with secondary antibodies in 1% BSA/PBS at room temperature for 1 h. After four washes with PBST and one wash with PBS (10 min each), sections were incubated with Vector TrueVIEW autofluorescence quenching solution (Vector Laboratories, SP-8400-15) for 10 min at room temperature. Nuclei staining with DAPI or TO-PRO-3 (Thermo Fisher Scientific, T3605) and mounting with VECTASHIELD Vibrance Antifade Mounting Medium (Vector Laboratories, H-1700) was performed as recommended by Vector TrueVIEW autofluorescence quenching kit. Images were acquired using a Zeiss LSM710 confocal microscope. Display settings were adjusted based on negative staining controls (no primary or secondary antibodies) and applied similarly to every image using Zen 2 lite or Fiji software.

### Bulk RNA-sequencing and data analysis

Embryonic livers were isolated at E12.5 from *Sox17*^Δ*50/*Δ*50*^, Sox17^Δ50/GFPCre^ and littermate WT embryos. GBs were isolated from P21 *Sox17^mut5/mut5^* or *Sox17^+/+^* animals and placed into ice cold PBS. RNA was then isolated using a Promega Maxwell^®^ 16 LEV simplyRNA Purification Kit. RNA quality was assessed using an Agilent 2100 Bioanalyzer and only those samples with an RNA integrity number (RIN) 7 or above were used to produce cDNA libraries using a Low Input Library Prep Kit. Then 150 nucleotide paired-end reads were obtained by Novogene using an Illumina NovaSeq6000 instrument that resulted in at least 40 M raw sequencing reads per sample. The Spliced Transcripts Alignment to a Reference (STAR v2.6.0c) application (Dobin et al., 2013) was used to perform sequence alignments to the GRCm38/mm10 mouse genome reference and GENCODE comprehensive gene annotations (Release M17). DESeq2 was employed for additional sample-level quality control analysis and downstream pairwise comparisons (Love et al., 2014). Gene ontology analysis of differentially expressed genes was performed using Metascape (Zhou et al., 2019).

### Single cell RNA-seq

Timed matings between CD-1 WT mice or between *Sox17^Δ50/Δ50^* mice were performed. WT and *Sox17^Δ50/Δ50^* embryos were collected separately at E10.5 on ice-cold PBS. Posterior foregut and midgut regions of embryos were dissected by removal of embryonic head, heart, tail, limb buds, posterior and dorsal body trunk. Dissected embryonic tissues were pooled according to genotype and dissociated into single cells by incubating and regular pipetting in Accumax (MilliporeSigma, A7089) supplemented with DNase I (Thermo Fisher Scientific, AM2222) for 15 min at 37°C. WT and *Sox17^Δ50/Δ50^* cell samples were then stained separately using Cell Multiplexing Oligo (10x Genomics 3′ CellPlex Kit) according to manufacturer recommendations, counted and pooled together at 1:1 ratio in PBS containing 10% FBS on ice. After washing three times with PBS containing 10% FBS, Fc blocks for 15 min followed by Epcam-PE antibody staining for 30 min was performed on ice with pooled cells (see Table S7 for an antibody list and a dilution factor). Cells were washed and DAPI staining was performed 15 min before sorting to enrich live epithelial cells at Vanderbilt Flow Cytometry Shared Resource. Library preparation and sequencing was carried out by Vanderbilt Technologies for Advanced Genomics using Chromium Single Cell 3′ Reagent Kits v3.1. A NovaSeq 6000 device was used to acquire 150 bp paired-end reads. Base calling was performed by RTA (version 2.4.11; Illumina), and further analysis was carried out using 10x Genomics Cell Ranger software v3.0.2. Initial quality control filtration, demultiplexing, unsupervised clustering, and differential gene expression analysis used Seurat 4.

### Generation of definitive endoderm

Definitive endoderm generation was carried out as previously described (Trinh et al., 2022). Specifically, TL1 mESCs were differentiated to definitive endoderm using IDE2 (StemCell Technologies, 72522), a small molecule, as previously described (Borowiak et al., 2009) with minor modifications. MEF-depleted mESCs were seeded at 5000 cells/cm^2^ overnight in mESC complete medium. After 24 h (start day 1), mESC complete medium was replaced with advanced RPMI 1640 medium (Thermo Fisher Scientific, 12633-012) supplemented with 2 mM L-glutamine, 0.2% heat-inactivated FBS and 5 μM IDE2.

### Luciferase reporter assay

Plasmids containing the WT TSS2 promoter, CR2, and plasmids containing the various mutations within CR2 were generated as previously described (Trinh et al., 2022). Test vectors, pGL2 Firefly positive control vector, pRL-SV40 *Renilla* control vector, and overexpression plasmids for Sox17 (pRP[Exp]-mCherry-CAG>mSox17[NM_001289464.1]); Sox9 (pRP[Exp]-mCherry-CAG>mSox9[NM_011448.4]); Sp1 (pN3-Sp1FL, Addgene plasmid #24543); Sp2 (pN3-Sp2FL, Addgene plasmid #107718); Sp3 (pN3-Sp3FL, Addgene #24541) (sequences found in Table S5) were transfected to cell culture at day 6 of differentiation using the standard protocol of Xfect Transfection Reagent Kit (Takara Bio, 631318). When applicable, transfected wells received 0.5 µg of the vectors for *Sox17*, *Sox9*, *Sp1*, *Sp2*, and *Sp3*, as well as 1 µg of the pGL2 Firefly positive control vector, and 0.01 µg of the pRL-SV40 *Renilla* control vector. Transfection media was replaced after 24 h with fresh differentiation media. Samples were collected 1 day later with passive lysis protocol of Dual-Luciferase Reporter Assay Kit (Promega, E1910) and luciferase activity was measured on Promega GloMax Discover Microplate Reader.

### Fluorescence-activated cell sorting

Recently dissected E9.5 mouse embryos were dissociated at 37°C for 10 min with Accumax solution containing 2 U/ml DNase I. On ice, cells were filtered through 35 μm strainer cap of a Falcon tube (Corning, 352235), quantified, pelleted by centrifugation at 1000 rpm (200 ***g***) for 3 min at 4°C and resuspended in flow cytometry staining buffer (R&D Systems, FC001) containing 2 U/ml DNase I (Flow/DNaseI buffer). Fc-blocking occurred for all samples with mouse IgG for 15 min, followed by incubation with fluorophore conjugated-antibodies in blocking solution for 30 min at RT (see Table S7 for an antibody list and a dilution factor). Stained cells were washed once with flow cytometry staining buffer and stained with DAPI in Flow/DNaseI buffer 15 min at room temperature, before being sorted into homogenization buffer (from Maxwell^®^ 16 LEV simplyRNA Purification Kits) at the Vanderbilt Flow Cytometry Shared Resource using a four-laser FACAria III.

### Graphical illustration, quantification and statistical analysis

Wherever applied, the performed statistical test, excluded samples, exact *n* number and what *n* represents are indicated in the figure legend. Processed ChIP-seq datasets were visualized using the UCSC genome browser. Sequence alignment in Fig. S7 was performed using Clustal Omega. Graphical illustrations in Figs 1, 6 and 7 were created with BioRender.com. Segmentation mask for SOX17^+^ epithelial nuclei was carried out using Ilastik (Berg et al., 2019) and single-nuclear signal intensity was quantified using FIJI. All other quantification visualization and statistics were carried out with GraphPad Prism 10 software. Results are shown as individual dots representing replications (details on replication types can be found in figure legends) and bars representing mean±s.e.m.

### Electrophoretic mobility shift assay

DNA probes contained a 38 bp region of *Sox17* promoter 2 spanning m4 and m5 regions. Three different probes were used: a WT promoter sequence, a Mut4 mutant containing a transversion mutation of the m4 region, and a Mut5 mutant containing a transversion mutation of the m5 region. Biotinylated DNA probes were made by annealing synthesized direct biotinylated and reverse unlabeled oligonucleotides. Competing cDNA probes were made by annealing synthesized unlabeled direct and reverse oligonucleotides (oligonucleotide sequences are in Table S6). HEK 293T cells were transfected with mouse *Sox17* (plasmid pRP[Exp]-mCherry-CAG>mSox17[NM_001289464.1]) using Xfect Transfection Reagent Kit. Cell nuclei were isolated 48 h after transfection and lysed in extraction buffer (500 mM KCl, 25 mM Hepes, 10% glycerol, 125 mM DTT, 1× protease inhibitors). We used 5 mg of total protein per EMSA assay. The nuclear lysate from untransfected cells was used as a control. EMSA was performed using the LightShift Chemiluminescent EMSA Kit (Thermo Fisher Scientific, 20148) according to the manufacturer's instructions. Briefly, target DNA probes were incubated with protein lysate with or without the addition of competing DNA probes (4 pM) in the binding reaction buffer [10 mM Tris HCl pH 8, 100 mM KCl, 1 mM DTT, 5 mM MgCl_2_, 50 ng/ml Poly (dI*dC), 0.05% NP40] for 20 min, and DNA-protein complexes were resolved by electrophoresis on 10% non-denaturing TBE polyacrylamide gel. After electrophoretic transfer to a nylon membrane (Zeta-Probe, Bio-Rad), biotin-labeled DNA was detected by staining with anti-biotin-HRP conjugated antibodies followed by a chemiluminescent HRP reaction (see Table S7 for an antibody list and a dilution factor). Visualization was carried out using Bio-Rad ChemiDoc MP Imaging System.

### Visualization of SOX17 binding data

SOX17 CUT&RUN data from the cXEN extra-embryonic endoderm was obtained from GSM6591690 (Pham et al., 2023). The BIGWIG file was then loaded into the UCSC genome browser GRCm38/mm10 and the *y*-axis was set from 0 to 1.7. SOX17 ChIP-seq data from human IPSCs differentiated to definitive endoderm was obtained from GSM5538057 (Mukherjee et al., 2022). The BIGWIG file was loaded into the UCSC genome browser GRCh37/hg19 and the *y*-axis was set from 0 to 75.

## Supplementary Material



10.1242/develop.203033_sup1 Supplementary information

Table S1. *Sox17^mut5/mut5^* gallbladder differential expression and GO term analysis. Complete differential expression and GO term analysis of the bulk RNA-seq shown in Fig. 5.

Table S5. Plasmid sequences used to study *Sox17* autoregulation. List of plasmids used in this study and their respective inserted sequences used in Fig. 7.
