# Supplementary material for: Positive autoregulation of Sox17 is necessary for gallbladder and extrahepatic bile duct formation
Source: Development. 2025 Jan 16;152(2):dev203033. doi: 10.1242/dev.203033 (PMC11829758; doi:10.1242/dev.203033)
Supplement: Supplementary information [file develop-152-203033-s1.pdf]

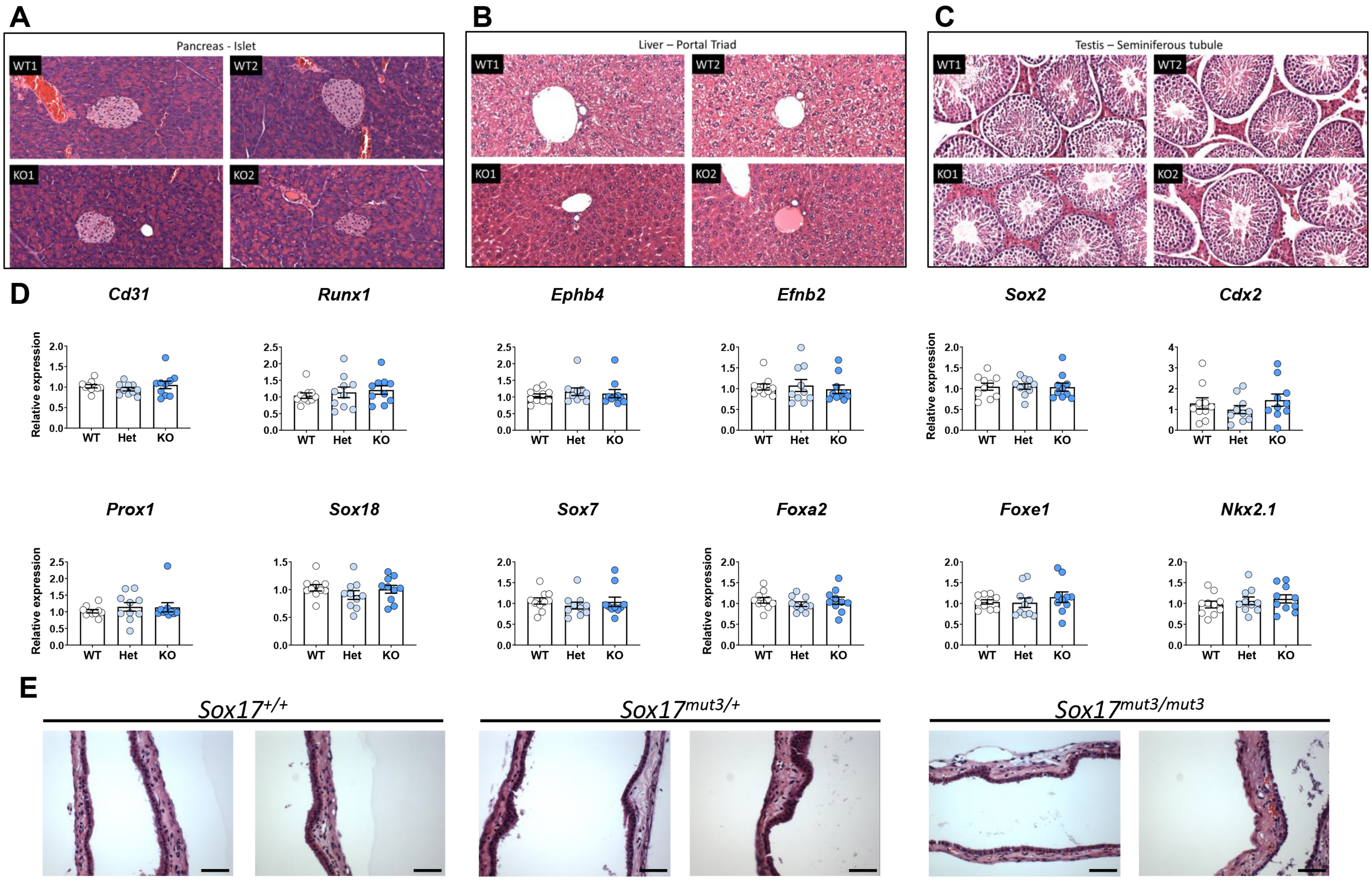

Fig. S1. Defects in *Sox17*$^{\Delta 50/\Delta 50}$ are limited to gallbladder development. (A-C) H&E staining of adult *Sox17*$^{\Delta 50/\Delta 50}$ pancreas, liver, and testes. (D) RT-qPCR analysis of various developmental marker genes in *Sox17*$^{\Delta 50/\Delta 50}$ (KO), *Sox17*$^{\Delta 50/+}$ heterozygous (Het) and wild type (WT) E9.5 embryos (n = 10). *Cd31*, endothelium (p = 0.9287). *Runx1*, hematopoietic progenitors (p = 0.6167). *Ephb4*, veins (p = 0.8831). *Efnb2*, arteries (p = 0.9336). *Sox2*, rostral endoderm (p = 0.9964). *Cdx2*, caudal endoderm (0.9006). *Prox1*, lymphatic system (p = 0.7371). *Sox18*, lymphatic vasculature (p = 0.9609). *Sox7*, vascular endothelium (p = 0.9878). *Foxa2*, pan-endoderm (p = 0.9953). *Foxe1*, lung and thyroid buds (0.7079). *Nkx2.1*, lung and thyroid buds (p = 0.5477). Data was obtained for each biological replicate by averaging three technical replicates and compared to wild-type littermates using Student's t-test. Values and error bars are mean ± s.e.m. (E) H&E staining of the gallbladder epithelium in P21 mut3 animals. Scale bar, 50 µM.

Development • Supplementary information

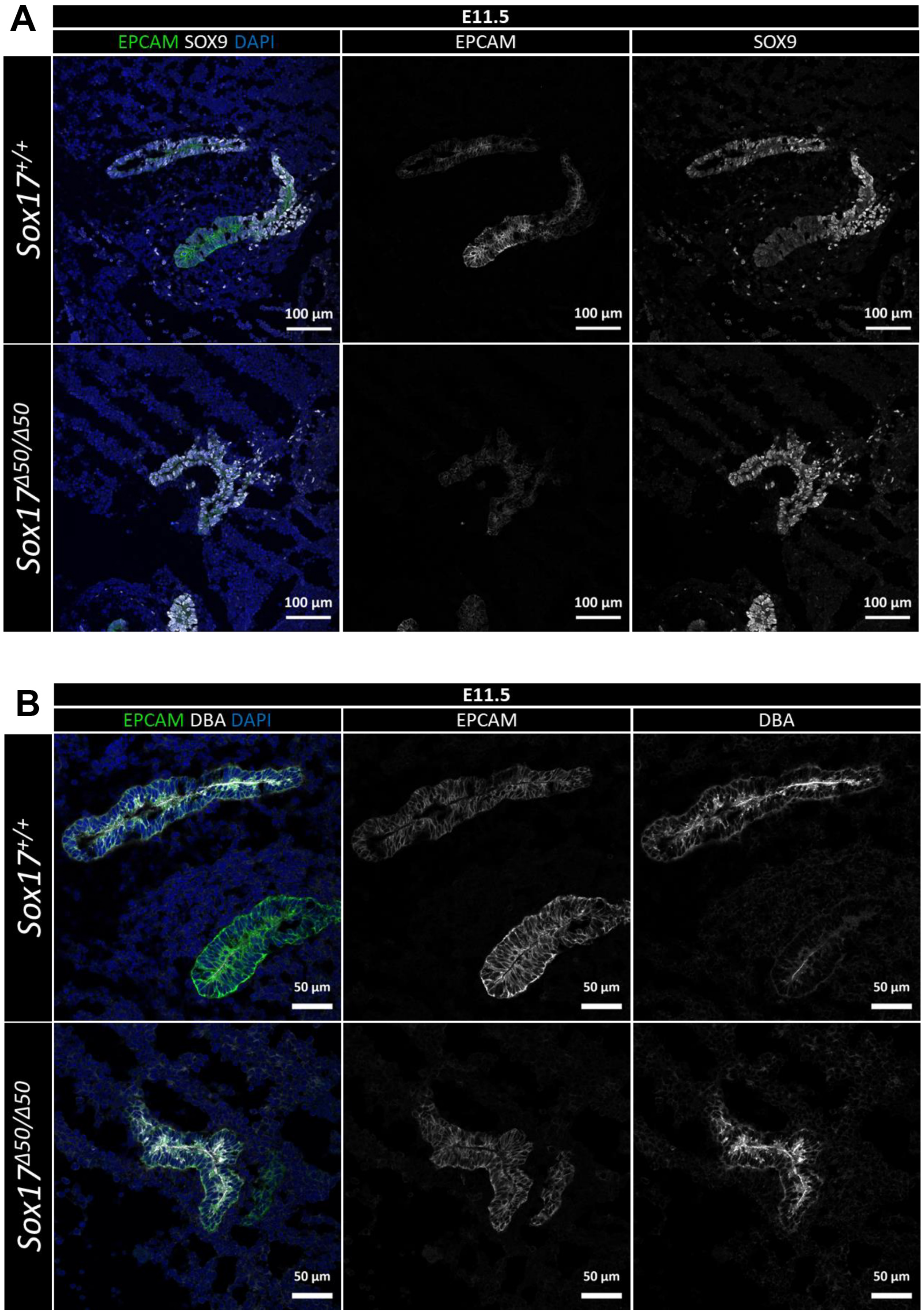

Fig. S2. Liver ductal development is not altered in *Sox17*$^{Δ50/Δ50}$ mutants despite the failure of the gallbladder to elongate. (A) Immuno-staining of EPCAM (epithelial) and SOX9 (ductal) in E11.5 *Sox17*$^{Δ50/Δ50}$ embryos. Scale bar, 100 µM. (B) Immuno-staining of EPCAM (epithelial) and DBA (ductal) in E11.5 *Sox17*$^{Δ50/Δ50}$ embryos. Scale bar, 50 µM.

Development • Supplementary information

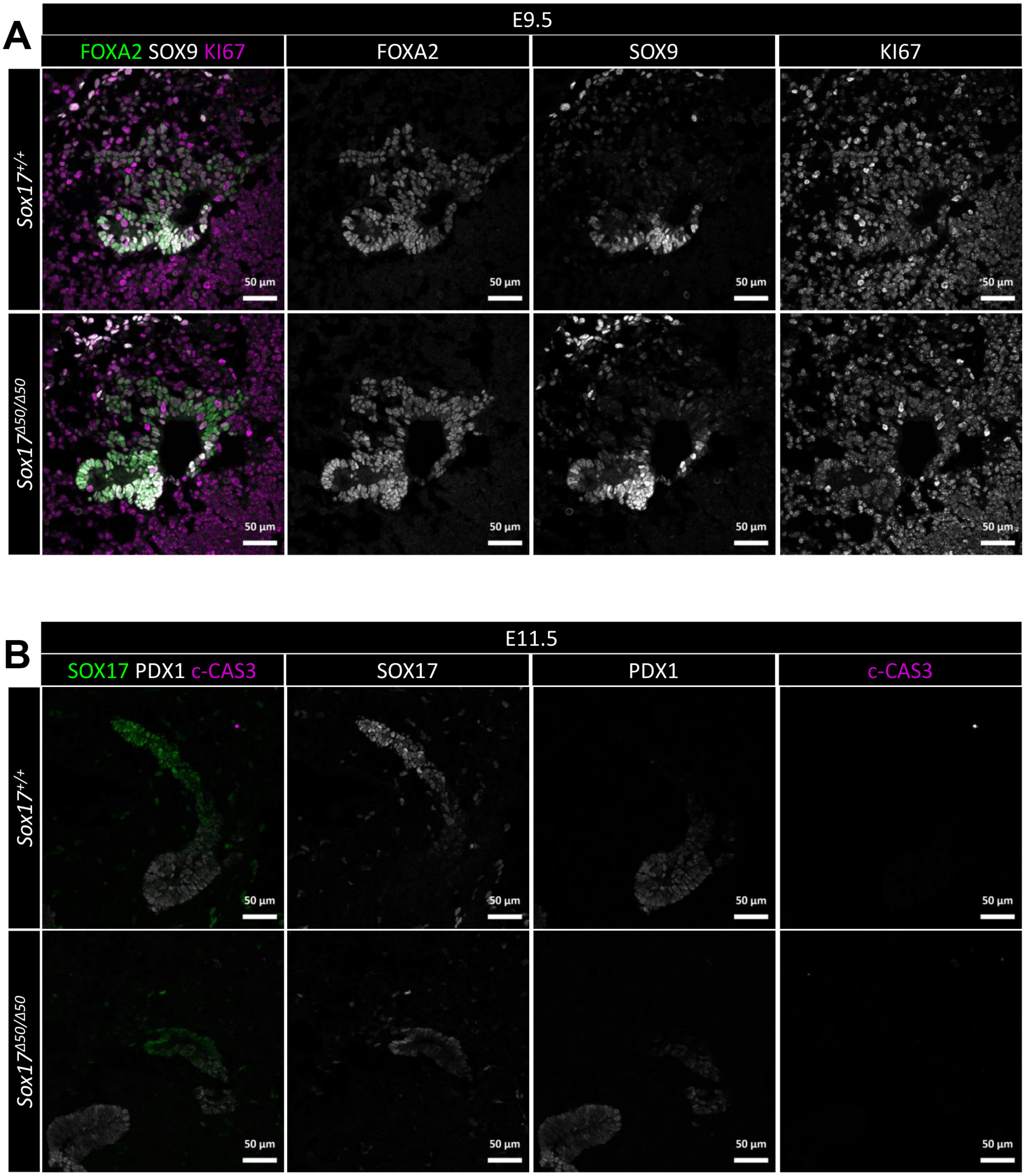

Fig. S3. Proliferation and cell death are not obviously changed in the developing biliary bud of *Sox17*$^{Δ50/Δ50}$ mutants. (A) Immuno-staining of FOXA2 (pan-endoderm), SOX9 (ductal) and KI67 (proliferation) in E9.5 *Sox17*$^{Δ50/Δ50}$ embryos. n = 1, 4 sections analyzed. (B) Immuno-staining of SOX17 (biliary), PDX1 (pancreas), and C-CAS3 (cell death) in E11.5 *Sox17*$^{Δ50/Δ50}$ embryos. n = 1, 11 sections analyzed.

Development • Supplementary information

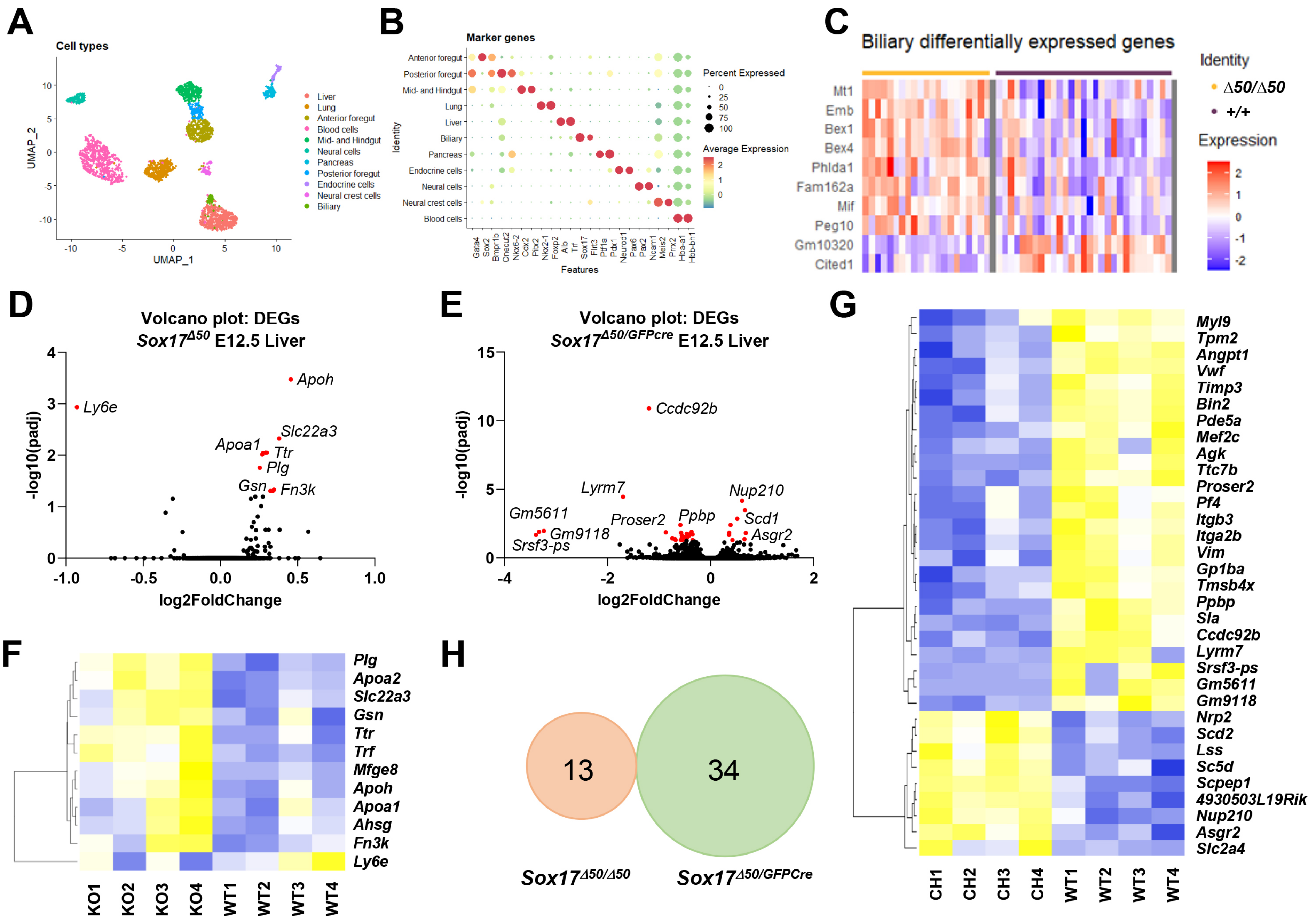

Fig. S4. scRNA-seq identifies 9 distinct cell types, including biliary cells, and transcriptomic profiles of *$Sox17^{\Delta50/\Delta50}$* and *$Sox17^{\Delta50/GFPCre}$* livers at E12.5 show no consistent differences. (A) UMAP of cells isolated for scRNAseq from EpCam+ FACS. n = 1 per genotype**.** (B) Marker gene expression correlates to each of the 9 distinct cell types recovered from scRNAseq. (C) Differentially up- (11) and down-regulated (2) genes within biliary cells from *$Sox17^{\Delta50/\Delta50}$* embryos at E10.5. (D) Bulk RNA-seq volcano plot of *$Sox17^{\Delta50/\Delta50}$* E12.5 livers showing differentially expressed genes (DEGs). (E) Bulk RNA-seq volcano plot of *$Sox17^{\Delta50/GFPCre}$* E12.5 livers showing DEGs. (F) Heatmap of DEGs identified within *$Sox17^{\Delta50/\Delta50}$* E12.5 livers. (G) Heatmap of DEGs in *$Sox17^{\Delta50/GFPCre}$* E12.5 livers. (H) Venn diagram of DEGs from both *$Sox17^{\Delta50/\Delta50}$* and *$Sox17^{\Delta50/GFPCre}$* E12.5 livers shows no overlap between two genotypes.

Development • Supplementary information

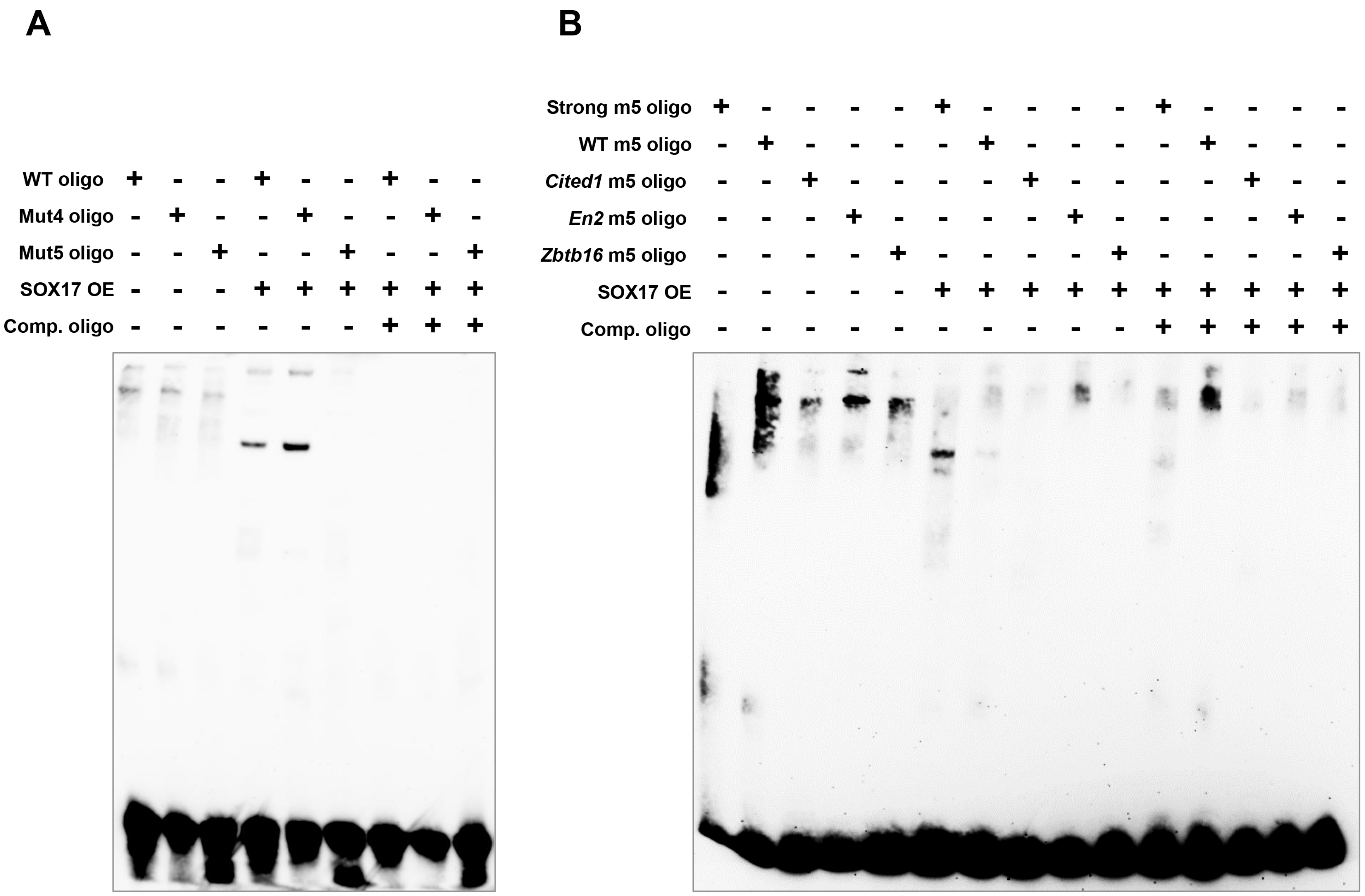

Fig. S5. Full image of EMSA blots presented in Figure 7D, 7E. (A) EMSA demonstrates direct binding of SOX17 at m5 region. The binding of SOX17 to WT (wild type) and mut4 mutant but not to mut5 mutant probe generates shifted bands. (B) EMSA demonstrates reduced binding at m5 region and other putative sub-optimal binding sites compared to an optimal SOX17 binding motif. SOX17 OE, *Sox17* overexpression lysate; com. probe, competing probe.

Development • Supplementary information

A **SOX17 binding near *CITED1***

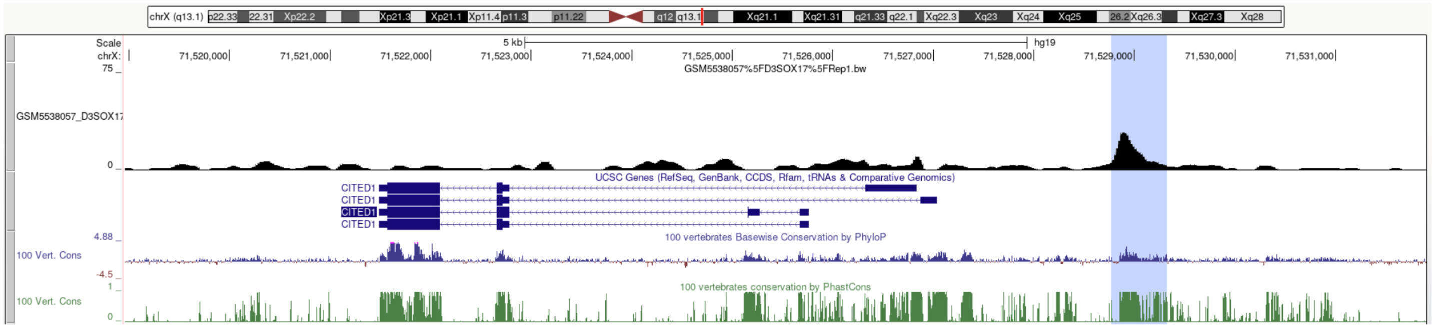

B **SOX17 binding near *EMB***

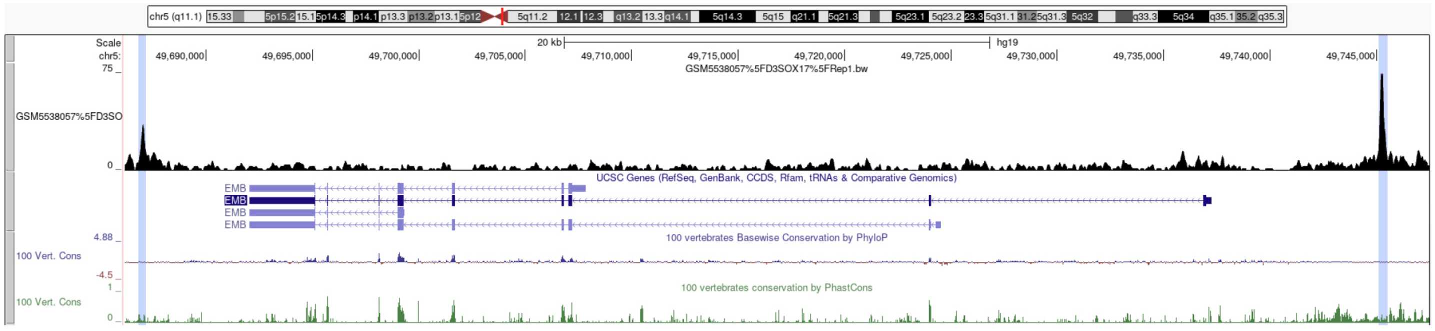

C **SOX17 binding near *EN2***

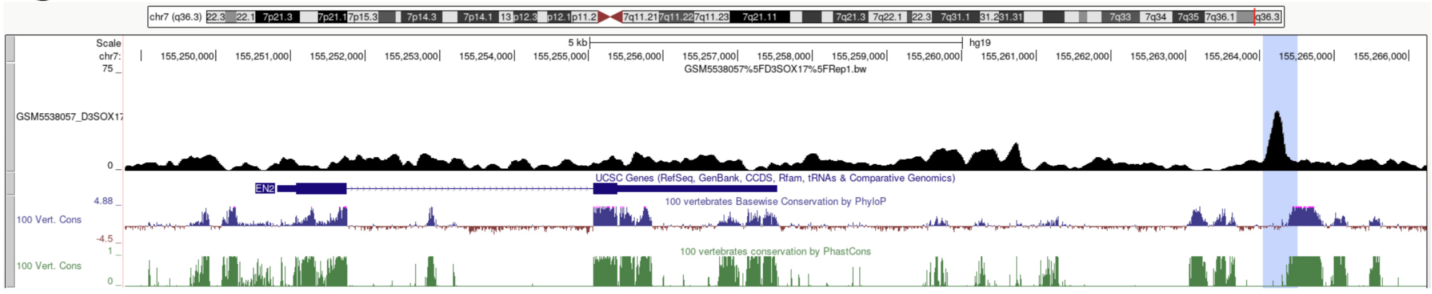

D

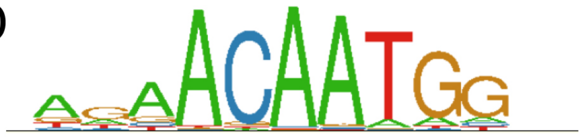

E

	Gene	SOX17 Binding Location (hg19)	SOX17 Motif Present	JASPAR Score
Δ50/Δ50 scRNA at E10.5	**Mt1**	chr16:56,655,922-56,656,221	AAGACAATGG	474
	**Emb**	chr5:49,745,097-49,745,499	GACACAATGG	400
		chr5:49,686,833-49,687,177	GAGACAATAG	388
	**Bex1 & Bex4**	chrX:102,356,522-102,357,018	GGGACAATTG	334
		chrX:102,371,380-102,371,839	AGAACAAAAG	397
	**Phlda1**	chr12:76,423,345-76,424,085	AAGACAATAG	387
		chr12:76,426,262-76,426,982	AGAACAAAGG	481
		chr12:76,405,298-76,405,658	ACGACAATGA	350
		chr12:76,407,857-76,408,240	ATAACAATAC	361
	**Mif**	chr22:24,242,364-24,242,782	CAAACAATAG	377
	**Peg10**	chr7:94,264,895-94,265,373	AAAACAATAG	464
		chr7:94,358,206-94,358,539	GAAACAAAGA	317

F

	Gene	SOX17 Binding Location (hg19)	SOX17 Motif Present	JASPAR Score
mut5/mut5 GB RNAseq at P21	**Cited1**	chrX:71,528,331-71,530,000	CTAACAATTG	330
		chrX:71,629,823-71,630,121	GGAACAATGG	541
	**En2**	chr7:155,264,041-155,264,499	GTGACAATGG	402
		chr7:155,185,292-155,185,697	GGAACAATAG	438
	**Tff3**	chr21:43,718,383-43,718,723	TCCACAATGG	354
	**Olfm4**	chr13:53,589,206-53,589,641	AGAACAAAGA	398
		chr13:53,535,398-53,535,781	ATTACAATAG	360
		chr13:53,525,387-53,525,986	AAAAAAATGA	321
	**Zbtb16**	chr11:113,928,113-113,928,500	GAAACAATGG	487
		chr11:113,948,496-113,949,020	AGGACAAAAG	335
		chr11:113,949,033-113,949,479	AAAACAAAGC	359
		chr11:113,985,904-113,986,281	CCAACAATAG	336
		chr11:114,018,749-114,019,041	AGGACAATGG	524
		chr11:114,090,642-114,091,001	AGAACAATGT	484
		chr11:114,103,341-114,103,721	AGGACAATGG	524

Fig. S6. SOX17 binding near *Sox17*-regulated genes. (A) SOX17 binding in hiPSCs differentiated to definitive endoderm near *CITED1*. SOX17 binding data, GEO series GSE182842, sample GSM5538057(Mukherjee et al., 2022). Viewing window GRCh37/hg19: ChrX:71,518,960-71,531,904. (B) SOX17 binding in hiPSCs differentiated to definitive endoderm near *EMB.* Viewing window GRCh37/hg19: Chr5:49,686,184-49,747,476. (C) SOX17 binding in hiPSCs differentiated to definitive endoderm near *EN2.* Viewing window GRCh37/hg19: Chr7:155,248,782-155,266,236. (D) SOX17 consensus motif as defined by the JASPAR database (Fornes et al., 2020). (E) Summary of SOX17 peaks nearby DEGs defined in the *Sox17*$^{\Delta 50/\Delta 50}$ E9.5 scRNA-seq dataset. SOX17 motifs present and JASPAR scores were identified using the JASPAR track of the UCSC genome browser GRCh37/hg19, and residues which are mismatched to the consensus sequence are denoted in red. Peaks were identified within a ± 105 kb window. (F) Summary of SOX17 peaks nearby select DEGs (Fig. 4E) defined in the *Sox17*$^{mut5/mut5}$ P21 gallbladder bulk RNA-seq dataset. SOX17 motifs present and JASPAR scores were identified using the JASPAR track of the UCSC genome browser GRCh37/hg19, and residues which are mismatched to the consensus sequence are denoted in red. Peaks were identified within a ± 105 kb window.

Development • Supplementary information

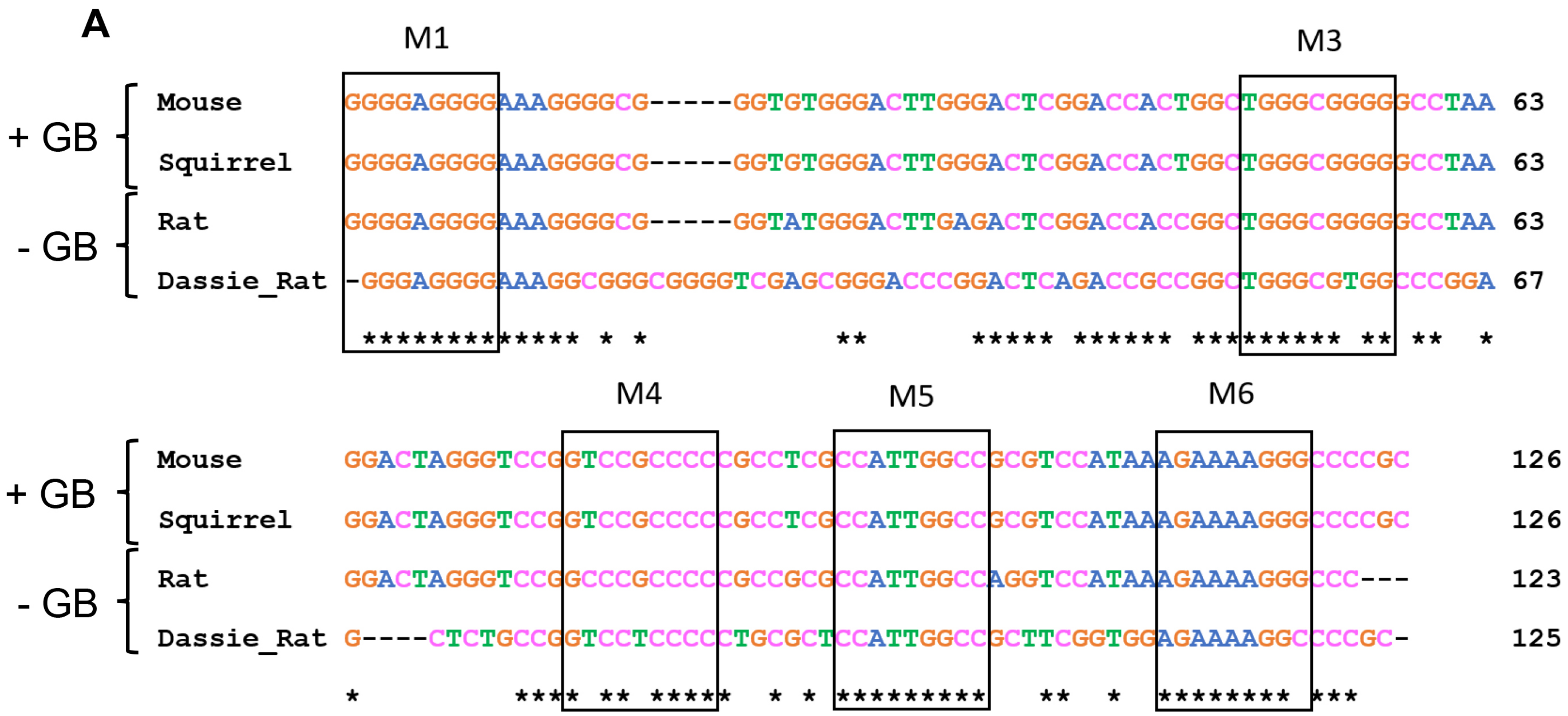

Fig. S7. Species-specific TSS2 promoter sequences do not correlate with the presence or absence of a gallbladder. (A) Sequence alignment of conserved region 2 (CRs) in the TSS2 promoter between two species with and two species without a gallbladder. Alignment was performed using Clustal Omega Multiple Sequence Alignment (https://www.ebi.ac.uk/jdispatcher/msa/clustalo). An * denotes a perfect sequence match between all four species. m1, m3, m4, m5, and m6 correspond to the CREs shown in Fig. 1A. GB, gallbladder. Mouse, *Mus musculus*. Squirrel, *Spermophilus tridecemlineatus*. Rat, *Rattus norvegicus*. Dassie Rat, *Petromus typicus*.

Table S1. ***Sox17*** $^{mut5/mut5}$ **gallbladder differential expression and GO term analysis.**

Complete differential expression and GO term analysis of the bulk RNA-seq shown in Fig. 5.

Available for download at
https://journals.biologists.com/dev/article-lookup/doi/10.1242/dev.203033#supplementary-data

Development • Supplementary information

Table S2. sgRNA & ssDNA used for mouse generation

Sequences of the sgRNA and ssDNA sequences utilized to create the *Sox17*$^{\Delta 50}$, *Sox17*^{mut5}, and *Sox17*^{mut3} lines used in this study.

Oligonucleotide	Sequence 5' → 3' (mutant sequences are bold and underlined)
Sox17_mut5 _gRNA	TTCTTTATGGACGCGGCCAA
Sox17_mut5 _ssDNA	GGGAAAGGGGCGGGTGTGGGACTTGGGACTCGGACCAC TGGCTGGGCGGGGGCCTAAGGACTAGGGTCCGGTCCGC CCCCGCCTCG**<u>AACGGTTAA</u>**GCGTCCATAAAGAAAAGGGC CCCGCGGCCCGGGGGGACACCCGCAGTGTCACTGGGTA GATCTCCAGCGCGGATTAGGCGAAGGG
Sox17_mut3 _gRNA	TGGGACTCGGACCACTGGCT
Sox17_mut3 _ssDNA	GGTGTCCCCCCGGGCCGCGGGGCCCTTTTCTTTATGGAC GCGGCCAATGGCGAGGCGGGGGCGGACCGGACCCTAGT CCTTAGGC**<u>AAAATAAAC</u>**GCCAGTGGTCCGAGTCCCAAGT CCCACACCCGCCCCTTTCCCCTCCCCCGGCAGTGTTCTGG CTACACCTGCCTTGGGGAAAACTG
Sox17_Δ50_ crRNA	TTCTTTATGGACGCGGCCAA

Table S3. PCR Primers

Complete list and sequences of PCR primers used in this study and their respective annealing temperatures.

Oligonucleotide	Purpose	Sequence 5' --> 3'	Annealing Temperature
Sox17-Δ128-For	Detection of Δ50, mut5, and mut3 alleles by PCR	CCAGAGGAACTCGTAAAGCTG	64°C
Sox17-Δ128-Rev1		GAATTTCAGCCTTCCTATTTCC	
Sox17-S2	Detection of Sox17 GFPCre allele by PCR	CAGAGGTATGCAGATCTCTGT	57°C
Sox17-3'UTR		CATTCTGGTCAACATGTAAGGT	
mSox17_long_F	Analysis of intron retention by PCR	CCAACACTCCTCCCAAAGTATCTATC	60°C
mSox17_long_R		GAAATGACGATTGAAAACAGTAGC	
mSox17_Exon4-5_F		CTTTATGGTGTGGGCCAAAGAC	60°C
mSox17_Exon4-5_R		GCTTCTCTGCCAAGGTCAACG	
mSox17_short_F		GGTTAGGTCTACCAGGAGAGC	60°C
mSox17_short_R		CTTCTCTGCCAAGGTCAACG	

Development • Supplementary information

Table S4. RT-qPCR Primers

Complete list and sequences of RT-qPCR primers used in this study.

Oligonucleotide	Sequence 5' --> 3'
mHNF4α-F	GTGAGCCTGGAGGATTACATC
mHNF4α-R	GGCATCGTGTTAGCAACAATG
mSox9-F	TGAAGAACGGACAAGCGGAG
mSox9-R	GATTGCCCAGAGTGCTCGC
mPdx1-F	CGGCTGAGCAAGCTAAGGTT
mPdx1-R	TGGAAGAAGCGCTCTCTTTGA
mSox17-a	GGTTAGGTCTACCAGGAGAGC
mSox17-c	CTTCTCTGCCAAGGTCAACG
mSox17-F1	CCAACACTCCTCCCAAAGTATCTATC
mSox17-R1	GAAATGACGATTGAAAACAGTAGC
mCited1-F	CCTGCACTTGATGTCAAGG
mCited1-R	CAGAGGAGCTAGTGGGAACTC
mSlc9a3-F	CTACCTGTCCTATCTGACCTC
mSlc9a3-R	CACTGGCCAGCATCTTCATAG
mEn2-F	GACAGCTCTCAAGCCAGC
mEn2-R	GCTTGTCCTCTTTGTTAGGG
mTff3-F	GATAGCTGCAGATTACGTTG
mTff3-R	GGATACTGGAGTCAAAGCAGC
mOlfm4-F	GAGGCACTTCTTGGGCAG
mOlfm4-R	CTAAGCGCTCCACTCTGTC
mZbtb16-F	GTCGAGCTTCCGGACAAC
mZbtb16-R	CAGTAAATGCATTCTCAGTCG
mCd31-F	CTTGTCGCAGTATCAGAATTTC
mCd31-R	CCTATGGCATGTCCTTTTATG
mRunx1-F	CTGCGGGGTTTCAACAGTTC
mRunx1-R	GAGCCCACTACCCTCTGTCTT
mEphb4-F	CTACGTCTCTAACCTCCCATCT
mEphb4-R	GCTGGTCACCCTTTCTCTTT
mEfnb2-F	GAACTGGGAGCGGCTTGG
mEfnb2-R	TTGTCCGGGTAGAAATTTGGAGTT
mSox2-F	GGCAGCTACAGCATGATGCAGGAGC
mSox2-R	CTGGTCATGGAGTTGTACTGCAGG
mCdx2-F	GAAACCTGTGCGAGTGGATG
mCdx2-R	CCAGCTCACTTTTCCTCCTG
mProx1-F	GTCATCACACCATCTGAGCC
mProx1-R	GGGTAGCGGGTGTAAAAGAAC
mSox18-F	CGACTGGCGCAACAAAATCC
mSox18-R	GGCCGGTACTTGTAGTTGGG
mSox7-F	CAAGGATGAGAGGAAACGTCTG
mSox7-R	CTTGTAGTTGGGGTAATCCTGC
mFoxa2-F	GGGAGCGGTGAAGATGGA
mFoxa2-R	TCATGTTGCTCACGGAGGAGTA
mFoxe1-F	TCATCACCGAGCGCTTCCCGTT
mFoxe1-R	GCGGCTGCATCGTGCATGTA
mNkx2.1-F	GCAACCTGGGCAACATGAG
mNkx2.1-R	CCATGTTCTTGCTCACGTCC

Table S5. Plasmid sequences used to study *Sox17* autoregulation.

List of plasmids used in this study and their respective inserted sequences used in Fig. 7.

Available for download at
https://journals.biologists.com/dev/article-lookup/doi/10.1242/dev.203033#supplementary-data

Development • Supplementary information

Table S6. EMSA Oligos

Sequences of the EMSA oligos used in Fig. 7 and Fig. S5.

Oligo Name	Sequence 5' to 3'	Modification
SOX/SP_WT_+_biotin (WT Oligo)	AGGGTCCGGTCCGCCCCCGCCTCGCCATTGGCCGCGTC	5'-Biotin
SOX/SP_WT_+	AGGGTCCGGTCCGCCCCCGCCTCGCCATTGGCCGCGTC	
SOX/SP_WT_-	GACGCGGCCAATGGCGAGGCGGGGGCGGACCGGACCCT	
SOX/SP_m4_+_biotin (Mut4 Oligo)	AGGGTCCGtgaataaaaCGCCTCGCCATTGGCCGCGTC	5'-Biotin
SOX/SP_m4_+	AGGGTCCGtgaataaaaCGCCTCGCCATTGGCCGCGTC	
SOX/SP_m4_-	GACGCGGCCAATGGCGAGGCGttttattcaCGGACCCT	
SOX/SP_m5_+_biotin (Mut5 Oligo)	AGGGTCCGGTCCGCCCCCGCCTCGaacggttaaGCGTC	5'-Biotin
SOX/SP_m5_+	AGGGTCCGGTCCGCCCCCGCCTCGaacggttaaGCGTC	
SOX/SP_m5_-	GACGCttaaccgttCGAGGCGGGGGCGGACCGGACCCT	
Pos_Sox17-F_B-FL	AGGGTCCGGTCCGCCCCCGCCTCGCCATTGTTCTGCGTC	5' Biotin
Pos_Sox17-F-FL	AGGGTCCGGTCCGCCCCCGCCTCGCCATTGTTCTGCGTC	
Pos_Sox17-R-FL	GACGCagaacaatggCGAGGCGGGGGCGGACCGGACCCT	
Cited1-F-B-FL	AGGGTCCGGTCCGCCCCCGCCTCGCTAACAATTGGCGTC	5' Biotin
Cited1-F-FL	AGGGTCCGGTCCGCCCCCGCCTCGCTAACAATTGGCGTC	
Cited1-R-FL	GACGCCAATTGTTAGCGAGGCGGGGGCGGACCGGACCCT	
Olfm4-F-B-FL	AGGGTCCGGTCCGCCCCCGCCTCGTCTTTGTTCTGCGTC	5' Biotin
Olfm4-F-FL	AGGGTCCGGTCCGCCCCCGCCTCGTCTTTGTTCTGCGTC	
Olfm4-R-FL	GACGCAGAACAAAGACGAGGCGGGGGCGGACCGGACCCT	
Zbtb16-F-B-FL	AGGGTCCGGTCCGCCCCCGCCTCGCTTTTGTCCTGCGTC	5' Biotin
Zbtb16-F-FL	AGGGTCCGGTCCGCCCCCGCCTCGCTTTTGTCCTGCGTC	
Zbtb16-R-FL	GACGCAGGACAAAAGCGAGGCGGGGGCGGACCGGACCCT	
En2-F-B-FL	AGGGTCCGGTCCGCCCCCGCCTCGGTGACAATGGGCGTC	5' Biotin
En2-F-FL	AGGGTCCGGTCCGCCCCCGCCTCGGTGACAATGGGCGTC	
En2-R-FL	GACGCCCATTGTCACCGAGGCGGGGGCGGACCGGACCCT	

Table S7. Antibodies

List of antibodies and their dilution factor used in this study.

Antibodies	Vendor	Catalog # / Identifier
Goat anti-SOX17 (1:100 dilution for immunofluorescence staining – IF)	R&D Systems	Cat# AF1924, RRID:AB_355060
Guinea pig anti-PDX1 (1:1000 dilution for IF)	Christopher V.E. Wright's lab	N/A
Rabbit anti-HNF4A (C11F12) (1:500 dilution for IF)	Cell Signaling Technology	Cat# 3113, RRID:AB_2295208
Rat anti-EPCAM (1:1000 for IF)	Santa Cruz	Cat# 53532
Rabbit anti-SOX9 (1:1000 for IF)	Millipore-Sigma	AB5535-25UG
Rabbit anti-FOXA2/HNF3B (D56D6) (1:400 dilution for IF)	Cell Signaling Technology	Cat# 8186, RRID:AB_10891055
Rabbit anti KI-67 (1:200 for IF)	Thermo Fisher Scientific	Cat# RM-9106-S1
Rabbit anti-cleaved caspase 3 (C-CAS3) (1:1000 dilution for IF)	Cell Signaling Technology	Cat #9661
PE-EpCam Rat Anti-Mouse CD326/EpCam (1/512ug per 10^6 cells for FACS)	BDBioscienc	Cat# 563477
Mouse CXCR4 APC-conjugated Antibody (0.25ug per 10^6 cells for FACS)	R&D Systems	Cat #MAB21651
APC Rat Anti-Mouse CD31 (1/128ug per 10^6 cells for FACS)	BDBioscience	Cat #551262
Rabbit anti TFF3 (1:1000 for IF)	Atlas Antibodies	Cat# HPA035464
Alexa Fluor 647 conjugated Donkey anti-Goat IgG (1:1000 dilution for IF)	Thermo Fisher Scientific	Cat# A21447, RRID:AB_2535864
Alexa Fluor 555 conjugated Donkey anti-Rabbit IgG (1:1000 dilution for IF)	Thermo Fisher Scientific	Cat# A31572, RRID:AB_162543
Alexa Fluor 488 conjugated Donkey anti-Goat IgG (1:1000 dilution for IF)	Thermo Fisher Scientific	Cat# A11055, RRID:AB_2534102
Alexa Fluor 647 conjugated Donkey anti-Guinea pig IgG (1:1000 dilution for IF)	Jackson Immunoresearch	Cat# 706-605-148, RRID: AB_2340476
Alexa Fluor 488 conjugated Donkey anti-Rat IgG (1:1000 dilution for IF)	Thermo Fisher Scientific	Cat# A21208, RRID:AB_2535794

Development • Supplementary information
