# Supplementary material for: Positive autoregulation of Sox17 is necessary for gallbladder and extrahepatic bile duct formation
Source: Development. 2025 Jan 16;152(2):dev203033. doi: 10.1242/dev.203033 (PMC11829758; doi:10.1242/dev.203033)
Supplement: Supplementary file 1 [file develop-152-203033_review_history.pdf]

Positive autoregulation of *Sox17* is necessary for gallbladder and extrahepatic bile duct formation

Linh T. Trinh, Ryan R. Finnel, Anna B. Osipovich, Jessica R. Musselman, Leesa L. Sampson, Christopher V.E. Wright and Mark A. Magnuson
DOI: 10.1242/dev.203033

Editor: James Wells

Review timeline

Original submission:	8 May 2024
Editorial decision:	25 June 2024
First revision received:	26 November 2024
Accepted:	17 December 2024

Original submission

First decision letter

MS ID#: DEVELOP/2024/203033

MS TITLE: Positive autoregulation of Sox17 is necessary for gallbladder and extrahepatic biliary duct formation

AUTHORS: Linh T Trinh, Ryan R Finnel, Anna B Osipovich, Jessica R Musselman, Leesa L Sampson, Christopher V.E. Wright, and Mark A Magnuson

I have now received all the referees' reports on the above manuscript, and have reached a decision. The referees' comments are appended below, or you can access them online: please go to BenchPress and click on the 'Manuscripts with Decisions' queue in the Author Area.

As you will see, two of the referees express considerable interest in your work, but have some significant criticisms and recommend a substantial revision of your manuscript before we can consider publication. Reviewer 1, while interested in the subject, commented that the the mechanistic advance over previous studies was modest. If you are able to revise the manuscript along the lines suggested, which would likely involve further experiments, I will be happy receive a revised version of the manuscript. Your revised paper will be re-reviewed by one or more of the original referees, and acceptance of your manuscript will depend on your addressing satisfactorily the reviewers' major concerns. Please also note that Development will normally permit only one round of major revision. If it would be helpful, you are welcome to contact us to discuss your revision in greater detail. Please send us a point-by-point response indicating your plans for addressing the referees' comments, and we will look over this and provide further guidance.

Please attend to all of the reviewers' comments and ensure that you clearly highlight all changes made in the revised manuscript. Please avoid using 'Tracked changes' in Word files as these are lost in PDF conversion. I should be grateful if you would also provide a point-by-point response detailing how you have dealt with the points raised by the reviewers in the 'Response to Reviewers' box. If you do not agree with any of their criticisms or suggestions please explain clearly why this is so.

Reviewer 1

Advance summary and potential significance to field

© 2024. Published by The Company of Biologists under the terms of the Creative Commons Attribution License (https://creativecommons.org/licenses/by/4.0/).

In this manuscript, Trinh & Finnel et al. report the generation of several mouse lines with deletions/mutations in different a cis-regulatory enhancers of Sox17.
In 2022 the authors reported that Sox17 has alternative transcription start sites with different enhancers resulting in a short and long form of the Sox17 mRNA with alternative 5Â'UTR. Here the authors demonstrate that deletion or inversion of a Sox17 binding site in one its own enhancers results in reduced expression of the short (but not long) mRNA isoform and a dose-dependent deficiency and metaplasia of gallbladder and extrahepatic biliary duct (GB&EHBD) formation. The authors provide some molecular analysis of the phenotype and data suggesting that Sox17 autoregulation controls the dose-response.

Overall, the mechanistic advances are rather modest. Sox17 is already known to control GB&EHBD development, with evidence for dose dependence (albeit not as convincing as this). Back in 2007, it was shown that Sox17 can regulate its own enhancers in Xenopus endoderm. If the authors could better determine the mechanisms of dose-dependent autoregulation and why the GB&EHBD is so sensitive while other tissues are not then the work would be acceptable for Development but that really would be a follow up mechanistic paper. In its current form, this study is largely a description of a phenotype.

Comments for the author

Major technical points:
1. Its not clear whether the different mRNA isoforms have different functions and whether the dose of Sox17 protein is sufficient to explain the phenotype. It appears that long-RNA form Sox17 is still expressed, and even upregulated, in Δ50 and mut5 animals. The authors don't rigorously quantify Sox17 protein levels, but immunostaining indicates that protein levels are decreased. The assumption that the short or long forms make the same amount of protein is not directly addressed. There may be differences in mRNA stability, translation and/or localization. It is not clear whether there is a one-to-one relationship between mRNA and protein levels for each isoform which impacts the author's interpretation of a simple dose-responsive phenotype.

2. The paper would be strengthened if the authors had some data to indicate why the GB&EHBD are uniquely sensitive to Sox17 dose.

3.The single-cell analysis of the phenotype at E9.5 is the right experiment, but as the authors state the analysis is underpowered with only one replicate and very few Sox17+ cells. This needs to be done properly with replicates for a rigorous differential expression analysis.

4.The analysis of P21 is complicated to interpret, since much of the phenotype and metaplasia may be indirect due to cholestasis as previously described. A more robust E9.5 analysis would be better explain the direct role of Sox17.

5.The enhancer-reporter assays are superficial and don't address dose-dependent effect as postulated in the discussion. Moreover, the finding that Sox17 can activate its own promximal endodermal enhancers was shown in Xenopus back in 2007 (PMID:17719026). There is an opportunity to test dose-dependent and non-dose-dependent enhancers here and to correlate this with different levels of SOX17 binding. It would also be good to do these analyses in mESCs differentiated into posterior foregut rather than DE.

<u>Reviewer 2</u>

Advance summary and potential significance to field

In this article Trinh et al. continue the work they published in 2022 on the regulation of Sox17 in mice. They had previously identified two important start sites (TSS1 & 2) associated with ci-regulatory regions (CR1 & CR2). While the associated transcripts encoded the same protein, TSS1 was specific to Sox17 expression in the vasculature, while CR2 controlled expression in both endothelial and endodermal cells. They had also used cell lines to show that CR2 was divided into 4 evolutionary conserved cis-regulatory DNA binding regions (CREs) called m1,3,4 & 5. In the present

© 2024. Published by The Company of Biologists under the terms of the Creative Commons Attribution License (https://creativecommons.org/licenses/by/4.0/).

manuscript, they generate three new mouse lines targeting m3 and m5, the two most important CREs as well as a fortuitously generated 50 bp deletion (within the previously studied 126 bp CR2). This allelic series combining heterozygous and homozygous alleles, as well as a full deletion results in different levels of Sox17 reduction in the emerging hepatobiliary-gallbladder region (35%, 50%, 60%, total loss) causing different defects in the endoderm and gall bladder. Beyond clarifying the regulatory regions of Sox17, the article reveals that the gallbladder and common bile duct are very sensitive to small reductions of Sox17. Additionally, the authors reveal a new Sox17 autoregulatory loop.

Strengths of the article:
- Very solid work and well written, convincingly documented and illustrated
- The investigation is extensive, combining exquisite mouse genetics gross observations, histology, modern single-cell and bulk transcriptome analysis, EMSA, reanalysis of ChIP-Seq data.
- An interesting case study that can be extended to show the important of protein expression
- Potential relevance to metaplasia observed in human conditions in the region Weaknesses of the article:
- As a follow-up study, some of the novelty emerged mostly in the first paper
- The importance of dosage has been shown for other Sox-family genes, as documented in the article and for others (for example Myc- https://www.nature.com/articles/414768a) or Pdx1. However, this is a first for this region.
- There are metaplasia phenotypes reported but no previous role of SOX17 reported in human. This being said, this article may trigger future studies.
- There is a very interesting discussion on the evolution of the hepato-biliary-gallbladder region but unfortunately the regions studied seem irrelevant.
- An interesting compensation of TSS1 when TSS2 is impaired is exciting but the mechanisms are not investigated. Given the breadth of the manuscript already, this would be for a next article.

Taken together this is a solid article that only needs minor improvement, as outlined below.

Comments for the author

Detailed comments:
- Page 4 paragraph 2, line 6 of the paragraph: the sentence starting with “While…” does not seem grammatically correct.
- Page 6, end of the section entitled “G&EHBD…”: in this instance and others, some conclusion wrapping-up each result section would help the reader particularly from other fields.
- Page 7, paragraph 2: The power of the single cell comparison of biliary cells is low due to the low amounts of cells, as noted later and addressed by further bulk sequencing. Is the sequencing depth also contributing to the limited sensitivity? Several of the up- and down-regulated transcripts identified by this method are not reported/discussed later in the bulk sequencing. It would be good to do so or reduce the report of results from the single-cell sequencing if there is little further validation of the findings.
- Page 9, last sentence of the section entitled “Marked…”: “… alterations in gallbladder epithelium that is similar to gastric and intestinal metaplasia and that has histological features previously associated with gallstones and malignancy.” The sentence lacks clarity with respect to which similarities and features. It is conveyed in a clearer way in the discussion.
- Page 12, paragraph 2: the last sentence should be toned-down as it is not formally shown that the sites are low-affinity binding sites. This is reported more carefully in the following paragraph.
- The observations from previously published SOX17 ChiP-Seq data in human even though in a different tissue type would be worth reporting as part of the results rather than just in the discussion. They are valuable. A question emerging is whether the Sox17 autoregulatory loop is also found in human. If not, it is worth commenting as well.

Reviewer 3

Advance summary and potential significance to field

© 2024. Published by The Company of Biologists under the terms of the Creative Commons Attribution License (https://creativecommons.org/licenses/by/4.0/).

In this manuscript, Magnuson and colleagues performed a thorough analysis of the regulation of Sox17 gene expression in mouse endoderm development. They used CRISPR/Cas9 gene editing to insert a series of promoter-proximal mutations near the Sox17 transcription start site (TSS) 2 and then examined the development of the hepatopancreatobiliary system. Both the gallbladder and cystic duct (part of the EHBD) were found to be exquisitely sensitive to the expression levels of Sox17. Furthermore, the authors identified a SOX17-binding cis-regulatory element within the TSS2 promoter and showed a SOX17-dependent autoregulatory loop, which would be responsible to boost Sox17 expression above a critical threshold concentration for proper gallbladder formation. Overall, this study is well-conducted and unveils another interesting example of TF dosage effects within the members of the SOX family.

Comments for the author

I have few concerns that need to be addressed and suggestions to improve the manuscript before it can be accepted for publication.
1) Throughout the study, the authors refer to the relevance of Sox17 expression levels for both gallbladder and EHBD development. However, this conclusion is not fully supported by the evidence presented, since only the cystic duct seems to be affected in the Sox17 mutant lines apart from the gallbladder. The analysis should be extended to the entire EHBD tree, not only to the cystic duct, and carried out at the histological level in addition to the gross morphology. Otherwise, the authors should revise the text and remove any reference to EHBD defects.
2) The intestinal or gastric metaplasia observed in Sox17mut5 line is interesting and deserves further analysis. Are the markers examined enough to conclude on the type of metaplasia / identity acquisition? Actually, the epithelium organization shown in Fig. 5 looks very similar to the EHBD tissue organization, including mucinous and peribiliary glands which are also positive for Epcam and TFF3 (see doi: 10.1002/hep.31252).
3) The authors performed an EMSA assay to show direct binding of Sox17 to the m5-CRE DNA in HEK 293 cells upon ectopic expression of Sox17. The same assay should be carried out in a more relevant cell model, for example using the mESC differentiation system, which seems to be established in their lab., to induce endogenous Sox17 expression or alternatively employing a Cut&Run assay.
4) All datasets (including the sc-transcriptomics) should be available as open-source tools. The access numbers to the EBI ArrayExpress<Biostudies> repository, which are included in the manuscript, do not seem to work.

First revision

<u>Author response to reviewers' comments</u>

Reviewer 1:

In this manuscript, Trinh & Finnel et al. report the generation of several mouse lines with deletions/mutations in different a cis-regulatory enhancers of Sox17. In 2022 the authors reported that Sox17 has alternative transcription start sites with different enhancers resulting in a short and long form of the Sox17 mRNA with alternative 5'UTR. Here the authors demonstrate that deletion or inversion of a Sox17 binding site in one its own enhancers results in reduced expression of the short (but not long) mRNA isoform and a dose-dependent deficiency and metaplasia of gallbladder and extrahepatic biliary duct (GB&EHBD) formation. The authors provide some molecular analysis of the phenotype and data suggesting that Sox17 autoregulation controls the dose-response.

Overall, the mechanistic advances are rather modest. Sox17 is already known to control GB&EHBD development, with evidence for dose dependence (albeit not as convincing as this). Back in 2007, it was shown that Sox17 can regulate its own enhancers in Xenopus endoderm. If the authors could better determine the mechanisms of dose-dependent autoregulation and why the GB&EHBD is so sensitive while other tissues are not then the work would be acceptable for Development, but that really would be a follow up mechanistic paper. In its

© 2024. Published by The Company of Biologists under the terms of the Creative Commons Attribution License (https://creativecommons.org/licenses/by/4.0/).

current form, this study is largely a description of a phenotype.

We agree that Sox17 was already known to control GB&EHBD formation. The three discrete mutations in the TSS2 promoter which we derived and studied show how this is achieved.

Comments for the Author:
Major technical points:

1. Its not clear whether the different mRNA isoforms have different functions and whether the dose of Sox17 protein is sufficient to explain the phenotype. It appears that long-RNA form Sox17 is still expressed, and even upregulated, in Δ50 and mut5 animals. The authors don't rigorously quantify Sox17 protein levels, but immunostaining indicates that protein levels are decreased. The assumption that the short or long forms make the same amount of protein is not directly addressed. There may be differences in mRNA stability, translation and/or localization. It is not clear whether there is a one-to-one relationship between mRNA and protein levels for each isoform which impacts the author's interpretation of a simple dose-responsive phenotype.

In Fig 3B, we quantified SOX17 protein levels via immuno-fluorescence in $\textit{Sox17}^{\square 50/\square 50}$ mice and showed a ~42% reduction in SOX17 protein levels. This closely approximates the ~50% reduction of the *Sox17* short mRNA determined by RT-qPCR. We believe that this data establishes a close correspondence between RNA produced from the TSS2 promoter, and protein levels.

We have previously shown that the TSS1-derived long mRNA is produced in vascular endothelium, and not endoderm, whereas the TSS2-derived short mRNA is produced in both the vascular endothelium and endoderm (Trinh et al., 2022) and that deletion of the TSS1 promoter resulted in only a minor phenotype. In this current study, the mutations we have introduced in the TSS2 promoter cause reductions in the short RNA form that correlate closely with impairment in the endoderm-derived biliary primordium whereas our measurements of the long mRNA do not show a correlation. Based on this data, as well as the lack of a TSS1 promoter in humans, we think that the regulation occurring at the TSS2 promoter is what determines GB&EHBD formation.

2. The paper would be strengthened if the authors had some data to indicate why the GB&EHBD are uniquely sensitive to Sox17 dose.

We agree and have added two additional pieces of data. First, we obtained two additional datapoints (from $\textit{Sox17}^{\Delta 50/+}$ and $\textit{Sox17}^{mut5/+}$ mice) for the *Sox17* expression/phenotype correlation shown in Figs 2B and 2D. Second, we performed an EMSA analysis (Fig 7E) that compared an optimal *Sox17* binding motif with that found in the m5 region of the *Sox17* TSS2 promoter, as well as those nearby several other genes regulated by *Sox17*. The SOX17 binding site within the TSS2 promoter and at other selected sites have a greatly reduced amount of SOX17 binding when compared to the optimal SOX17 motif. We believe that this data strengthens our assertion that SOX17 binding sites important for development of the GB&EHBD are suboptimal (low affinity) and that they require high levels of SOX17 expression to enhance transcription.

3. The single-cell analysis of the phenotype at E9.5 is the right experiment, but as the authors state the analysis is underpowered with only one replicate and very few Sox17+ cells. This needs to be done properly with replicates for a rigorous differential expression analysis.

We agree, and delayed resubmission for several months while we attempted to provide better data. After consulting with our NGS core and discussing our needs, they recommended we try an alternate experimental pipeline. Unfortunately, and despite considerable effort on our part, the results were totally unusable. We concluded that it will likely require six months or more to establish a workable pipeline, and then to collect and analyze the desired data. Given that we had already delayed resubmission for 3 months, we decided to proceed with resubmission.
Since we agree with the reviewer that the current differential expression analysis is

© 2024. Published by The Company of Biologists under the terms of the Creative Commons Attribution License (https://creativecommons.org/licenses/by/4.0/).

suboptimal, we moved the results we had to a supplemental figure and now only emphasize the changes in *Sox17* and *Cited1*, both of which were validated in our *Sox17*$^{mut5/mut5}$ gallbladder bulk RNA-seq dataset. We remind the reviewer that the cell population of interest is only 2% or so of the less than 5000 EpCam+ cells present in embryos at E10.5.

4. The analysis of P21 is complicated to interpret, since much of the phenotype and metaplasia may be indirect due to cholestasis as previously described. A more robust E9.5 analysis would be better explain the direct role of Sox17.

We performed additional H&E staining the common bile duct of the *Sox17*$^{\Delta50/+}$ animals at P21. Animals of this genotype have a similar gallbladder hypoplasia phenotype as the *Sox17*$^{mut/5mut5}$ and do similarly not exhibit cholestasis. The analysis revealed no abnormalities in the common bile duct (see below).

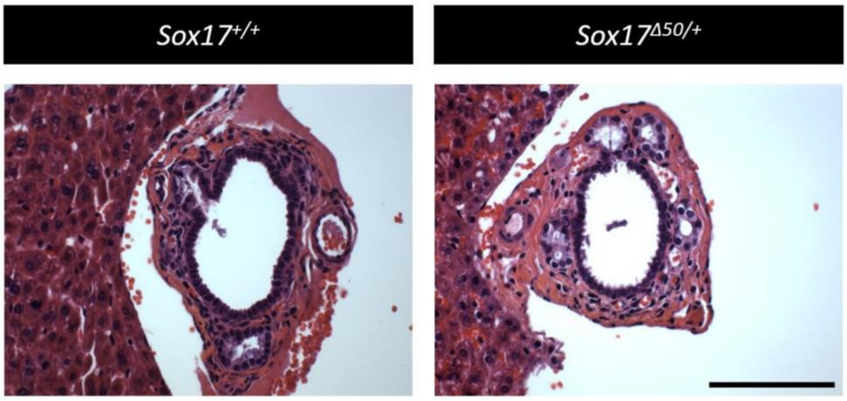

Figure: H&E staining of the common bile duct in P21 *Sox17*$^{+/+}$ and *Sox17*$^{\Delta50/+}$ animals. Scale bar, 100 uM.

5. The enhancer-reporter assays are superficial and don't address dose-dependent effect as postulated in the discussion. Moreover, the finding that Sox17 can activate its own proximal endodermal enhancers was shown in Xenopus back in 2007 (PMID:17719026). There is an opportunity to test dose-dependent and non-dose-dependent enhancers here and to correlate this with different levels of SOX17 binding. It would also be good to do these analyses in mESCs differentiated into posterior foregut rather than DE.

Thank you for bringing the 2007 study to our attention. The prior study describes *Sox17* autoregulation at enhancer elements located 9 kb upstream of *Sox17*. While both the species and element location are different, we agree that the prior study is complimentary and now cite it. The *Xenopus* study suggests autoregulation at the enhancer level while our study in mice indicates that the autoregulation occurs at the promoter level.

Reviewer 2:

In this article Trinh et al. continue the work they published in 2022 on the regulation of Sox17 in mice. They had previously identified two important start sites (TSS1 & 2) associated with ci- regulatory regions (CR1 & CR2). While the associated transcripts encoded the same protein, TSS1 was specific to Sox17 expression in the vasculature, while CR2 controlled expression in both endothelial and endodermal cells. They had also used cell lines to show that CR2 was divided into 4 evolutionary conserved cis-regulatory DNA binding regions (CREs) called m1,3,4 & 5. In the present manuscript, they generate three new mouse lines targeting m3 and m5, the two most important CREs as well as a fortuitously generated 50 bp deletion (within the previously studied 126 bp CR2). This allelic series combining heterozygous and homozygous alleles, as well as a full deletion results in different levels of Sox17 reduction in the emerging hepatobiliary-gallbladder region (35%, 50%, 60%, total loss) causing different defects in the endoderm and gall bladder. Beyond clarifying the regulatory regions of Sox17,

© 2024. Published by The Company of Biologists under the terms of the Creative Commons Attribution License (https://creativecommons.org/licenses/by/4.0/).

the article reveals that the gallbladder and common bile duct are very sensitive to small reductions of Sox17. Additionally, the authors reveal a new Sox17 autoregulatory loop.

Strengths of the article:
-Very solid work and well written, convincingly documented and illustrated.

Thank you.

-The investigation is extensive, combining exquisite mouse genetics, gross observations, histology, modern single-cell and bulk transcriptome analysis, EMSA, reanalysis of ChIP-Seq data.

Thank you.

-An interesting case study that can be extended to show the important of protein expression

-Potential relevance to metaplasia observed in human conditions in the region.

Weaknesses of the article:
-As a follow-up study, some of the novelty emerged mostly in the first paper.

Our prior study laid the groundwork for the current analysis. We would not have known which sequences to mutate, nor would we have been motivated to do so, without it.

-The importance of dosage has been shown for other Sox-family genes, as documented in the article and for others (for example Myc-https://www.nature.com/articles/414768a) or Pdx1. However, this is a first for this region.

-There are metaplasia phenotypes reported but no previous role of SOX17 reported in human. This being said, this article may trigger future studies.

We agree.

-There is a very interesting discussion on the evolution of the hepato-biliary-gallbladder region but unfortunately the regions studied seem irrelevant.

We agree.

-An interesting compensation of TSS1 when TSS2 is impaired is exciting but the mechanisms are not investigated. Given the breadth of the manuscript already, this would be for a next article.

Given the lack of a clear trend, that the TSS1 promoter is not present in humans, and that deletion of the TSS1 promoter region in mice yielded only a subtle phenotype, we have focused our studies for now on the TSS2 region. This point is further discussed in response to point 1 by reviewer 1.

Taken together this is a solid article that only needs minor improvement, as outlined below.

Comments for the Author:
Detailed comments:
-Page 4 paragraph 2, line 6 of the paragraph: the sentence starting with “While…” does not seem grammatically correct.

We agree and have fixed this sentence.

-Page 6, end of the section entitled “G&EHBD…”: in this instance and others, some conclusion wrapping-up each result section would help the reader, particularly from other fields.

We agree and have added additional concluding sentences to multiple sections.

© 2024. Published by The Company of Biologists under the terms of the Creative Commons Attribution License (https://creativecommons.org/licenses/by/4.0/).

-Page 7, paragraph 2: The power of the single cell comparison of biliary cells is low due to the low amounts of cells, as noted later and addressed by further bulk sequencing. Is the sequencing depth also contributing to the limited sensitivity? Several of the up- and down-regulated transcripts identified by this method are not reported/discussed later in the bulk sequencing. It would be good to do so or reduce the report of results from the single-cell sequencing if there is little further validation of the findings.

The very low number of cells we obtained prevented us from obtaining more robust data. As described in our response to point 3 by reviewer 1, we attempted to improve upon the current scRNA analysis but have not yet succeeded.

Page 9, last sentence of the section entitled “Marked…”: “… alterations in gallbladder epithelium that is similar to gastric and intestinal metaplasia, and that has histological features previously associated with gallstones and malignancy.” The sentence lacks clarity with respect to which similarities and features. It is conveyed in a clearer way in the discussion.

We have clarified this sentence using similar language as described in the discussion.

Page 12, paragraph 2: the last sentence should be toned-down as it is not formally shown that the sites are low-affinity binding sites. This is reported more carefully in the following paragraph.

We have adjusted our wording to soften our claim.

-The observations from previously published SOX17 ChiP-Seq data in human, even though in a different tissue type would be worth reporting as part of the results rather than just in the discussion. They are valuable. A question emerging is whether the Sox17 autoregulatory loop is also found in human. If not, it is worth commenting as well.

We moved the results for the human SOX17 ChiP-Seq from the discussion to the results. Thank you for this suggestion.

To determine whether the same autoregulatory loop might also exist in humans, we found publicly reported SOX17 ChIP data, shown below, that may suggest an autoregulatory loop may be found in humans (Luo et al., 2023) ; GSE213394). We have added a comment to the discussion and have cited our previous paper which indicated the TSS2 promoter is also present in humans.

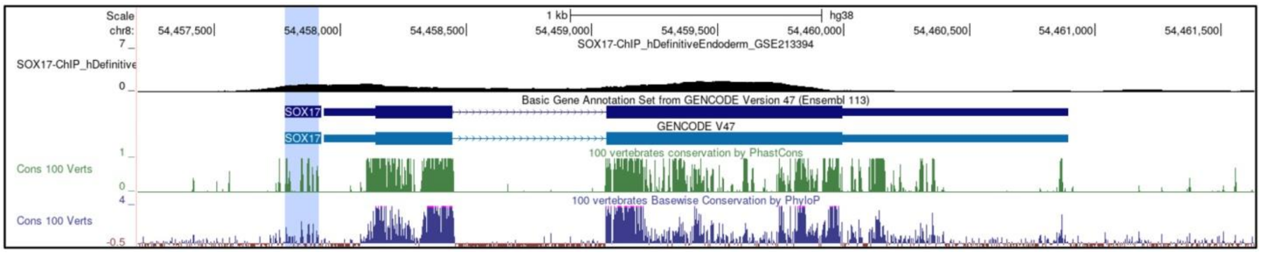

Figure: Human *SOX17* with SOX17 ChIP from GSE213394. Viewing window, GRCh38/hg38 chr8:54,457,196-54,461,632. The TSS2 promoter region is indicated as the blue highlight. The TSS2 promoter location in humans was identified using the UCSC Genome Browser LiftOver tool and is found at GRCh38/hg38 chr8:54457784-54457919.

Reviewer 3:

In this manuscript, Magnuson and colleagues performed a thorough analysis of the regulation of Sox17 gene expression in mouse endoderm development. They used CRISPR/Cas9 gene editing to insert a series of promoter-proximal mutations near the Sox17 transcription start site (TSS) 2 and then examined the development of the hepatopancreatobiliary system. Both

© 2024. Published by The Company of Biologists under the terms of the Creative Commons Attribution License (https://creativecommons.org/licenses/by/4.0/).

the gallbladder and cystic duct (part of the EHBD) were found to be exquisitely sensitive to the expression levels of Sox17. Furthermore, the authors identified a SOX17-binding cis-regulatory element within the TSS2 promoter and showed a SOX17-dependent autoregulatory loop, which would be responsible to boost Sox17 expression above a critical threshold concentration for proper gallbladder formation.

Overall, this study is well-conducted and unveils another interesting example of TF dosage effects within the members of the SOX family.

Thank you.

I have few concerns that need to be addressed and suggestions to improve the manuscript before it can be accepted for publication.

1) Throughout the study, the authors refer to the relevance of Sox17 expression levels for both gallbladder and EHBD development. However, this conclusion is not fully supported by the evidence presented, since only the cystic duct seems to be affected in the Sox17 mutant lines apart from the gallbladder. The analysis should be extended to the entire EHBD tree, not only to the cystic duct, and carried out at the histological level in addition to the gross morphology. Otherwise, the authors should revise the text and remove any reference to EHBD defects.

In addition to the cystic duct, we have also identified abnormalities in the common bile duct due to decreases in *Sox17* expression. The common bile duct becomes constricted causing biliary atresia in *Sox17*$^{\Delta 50/GFPCre}$ compound heterozygous animals (Fig 6C). When surveying the common bile duct in *Sox17*$^{\Delta 50/+}$ animals, we do not see any histological defects (see reviewer 1, point 4). While most of our mutants only exhibit defects within the gallbladder or cystic duct, the biliary atresia we observed in the *Sox17*$^{\Delta 50/GFPCre}$ animals indicates that *Sox17* is critical not only for the GB and cystic duct, but also for the EHBD tree.

2) The intestinal or gastric metaplasia observed in Sox17mut5 line is interesting and deserves further analysis. Are the markers examined enough to conclude on the type of metaplasia / identity acquisition? Actually, the epithelium organization shown in Fig. 5 looks very similar to the EHBD tissue organization, including mucinous and peribiliary glands which are also positive for Epcam and TFF3 (see doi: 10.1002/hep.31252).

Thank you for the thoughtful question. It has been shown that gallbladder epithelium does not contain goblet cells (Koga, 1973) or peribiliary glands (Matsubara et al., 2020). To confirm their absence, we sought to identify marker genes for each and survey their transcriptomic dynamics. By further probing the RNA-seq data from the *Sox17*$^{mut5/mut5}$ gallbladders, we identified two additional markers of goblet cells, *Agr2* and *Spink4* (Hensel et al., 2024), both of which were upregulated (*Agr2*, p-adj = 0.0316. *Spink4*, p-adj = 3.32E-07). Additionally, after educating ourselves a bit more and surveying the literature, we found that two markers of peribiliary glands, *Axin2* and *Muc6*, were unchanged in our dataset (*Axin2*, p-adj = 0.99903. *Muc6*, p-adj 0.513447) (Singh et al., 2023). Lastly, using the paper you cited above, we surveyed the genes that are representative of the EHBD cluster shown in Fig 7F (Rimland et al., 2021). We found that 7 of the 9 genes were unchanged in our dataset (see below). The two genes within the EHBD markers that were changed were *Tff3*, which is predominantly expressed in and serves as a marker of intestinal goblet cells (Yang et al., 2022), as well as *Plk1*. *Plk1* is highly expressed during mitosis and is considered by others as a master mitotic kinase that becomes oncogenic when dysregulated (Calvisi et al., 2023) (Yim and Erikson, 2014). This additional data points to the upregulation of TFF3 being indicative of intestinal metaplasia as opposed to the presence of ectopic peribiliary glands.

© 2024. Published by The Company of Biologists under the terms of the Creative Commons Attribution License (https://creativecommons.org/licenses/by/4.0/).

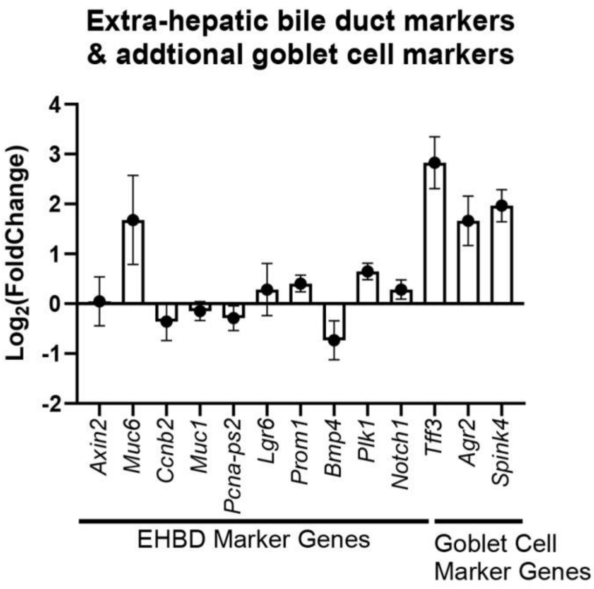

Tissue Marker	Gene Symbol	p-adj
EHBD	*Axin2*	0.99903
EHBD	*Muc6*	0.513447
EHBD	*Ccnb2*	0.880543
EHBD	*Muc1*	0.923243
EHBD	*Pcna-ps2*	0.813967
EHBD	*Lgr6*	0.958743
EHBD	*Prom1*	0.277178
EHBD	*Bmp4*	0.518632
EHBD	*Plk1*	0.006
EHBD	*Notch1*	0.710909
EHBD & Goblet Cells	*Tff3*	1.13E-05
Goblet Cells	*Agr2*	0.031592
Goblet Cells	*Spink4*	3.32E-07

3) The authors performed an EMSA assay to show direct binding of Sox17 to the m5-CRE DNA in HEK 293 cells upon ectopic expression of Sox17. The same assay should be carried out in a more relevant cell model, for example using the mESC differentiation system, which seems to be established in their lab., to induce endogenous Sox17 expression or alternatively employing a Cut&Run assay.

We agree with the value of identifying biologically-relevant SOX17 binding sites and measurement of their affinities. To do so, we have begun setting up a dTAG strategy in human PSCs. However, establishing this system and performing detailed studies will require time and effort that goes well beyond the current study.

4) All datasets (including the sc-transcriptomics) should be available as open-source tools. The access numbers to the EBI ArrayExpress repository, which are included in the manuscript, do not seem to work.

All datasets are now publicly accessible.

References

Calvisi, D. F., Boulter, L., Vaquero, J., Saborowski, A., Fabris, L., Rodrigues, P. M., Coulouarn, C., Castro, R. E., Segatto, O., Raggi, C. et al. (2023) 'Criteria for preclinical models of cholangiocarcinoma: scientific and medical relevance', *Nat Rev Gastroenterol Hepatol* 20(7): 462-480.
Hensel, I. V., Elias, S., Steinhauer, M., Stoll, B., Benfatto, S., Merkt, W., Krienke, S., Lorenz, H. M., Haas, J., Wildemann, B. et al. (2024) 'SLE serum induces altered goblet cell differentiation and leakiness in human intestinal organoids', *EMBO Mol Med* 16(3): 547-574. Koga, A. (1973) 'Electron microscopic observations on the mucous secretory activity of the human gallbladder epithelium', *Z Zellforsch Mikrosk Anat* 139(4): 463-71.
Luo, R., Yan, J., Oh, J. W., Xi, W., Shigaki, D., Wong, W., Cho, H. S., Murphy, D., Cutler, R., Rosen, B. P. et al. (2023) 'Dynamic network-guided CRISPRi screen identifies CTCF-loop- constrained nonlinear enhancer gene regulatory activity during cell state transitions', *Nat Genet* 55(8): 1336-1346.
Matsubara, T., Kozaka, K., Matsui, O., Nakanuma, Y., Uesaka, K., Inoue, D., Yoneda, N., Yoshida, K., Kitao, A., Yokka, A. et al. (2020) 'Peribiliary glands: development, dysfunction, related conditions and imaging findings', *Abdom Radiol (NY)* 45(2): 416-436.
Rimland, C. A., Tilson, S. G., Morell, C. M., Tomaz, R. A., Lu, W. Y., Adams, S. E., Georgakopoulos, N., Otaizo-Carrasquero, F., Myers, T. G., Ferdinand, J. R. et al. (2021) 'Regional Differences in Human Biliary Tissues and Corresponding In Vitro-Derived Organoids', *Hepatology* 73(1): 247-267.

© 2024. Published by The Company of Biologists under the terms of the Creative Commons Attribution License (https://creativecommons.org/licenses/by/4.0/).

Singh, S., Lian, Q., Budiman, T., Taketo, M. M., Simons, B. D. and Gupta, V. (2023) 'Heterogeneous murine peribiliary glands orchestrate compartmentalized epithelial renewal', *Dev Cell* 58(23): 2732-2745 e5.
Trinh, L. T., Osipovich, A. B., Sampson, L., Wong, J., Wright, C. V. E. and Magnuson, M. A. (2022) 'Differential regulation of alternate promoter regions in Sox17 during endodermal and vascular endothelial development', *iScience* 25(9): 104905.
Yang, Y., Lin, Z., Lin, Q., Bei, W. and Guo, J. (2022) 'Pathological and therapeutic roles of bioactive peptide trefoil factor 3 in diverse diseases: recent progress and perspective', *Cell Death Dis* 13(1): 62.
Yim, H. and Erikson, R. L. (2014) 'Plk1-targeted therapies in TP53- or RAS-mutated cancer', *Mutat Res Rev Mutat Res* 761: 31-39.

Second decision letter

MS ID#: DEVELOP/2024/203033

MS TITLE: Positive autoregulation of Sox17 is necessary for gallbladder and extrahepatic biliary duct formation

AUTHORS: Linh T Trinh, Ryan R Finnel, Anna B Osipovich, Jessica R Musselman, Leesa L Sampson, Christopher V.E. Wright, and Mark A Magnuson
ARTICLE TYPE: Research Article

I am happy to tell you that your manuscript has been accepted for publication in Development, pending our standard publication integrity checks.

Reviewer 1

Advance summary and potential significance to field

This will be a valuable contribution to our understanding of extra hepatic biliary development and SOX TF dose-dependent function.

Comments for the author

The authors have addressed the majority of my comments with new data, additional analysis or further explanation. I support publishing.

Reviewer 2

Advance summary and potential significance to field

The authors have revised the article addressing the questions of the reviewers (except for the difficult single-cell sequencing which was transferred to supplements, which is appropriate). This lead to a few figure and text modifications and some responses in the response letter justifying why a few of the comments did not justify changing the manuscript.

Comments for the author

The Sox17 peak in human looks very small. It would be beneficial to make this data available in supplements to give the reader the opportunity to review this and discuss it with a word of caution.

Reviewer 3

© 2024. Published by The Company of Biologists under the terms of the Creative Commons Attribution License (https://creativecommons.org/licenses/by/4.0/).

Advance summary and potential significance to field

In the revised manuscript, the authors have addressed all concerns raised by this reviewer.

Comments for the author

In the revised manuscript, the authors have addressed all concerns raised by this reviewer. The manuscript is now suitable for publication.

© 2024. Published by The Company of Biologists under the terms of the Creative Commons Attribution License (https://creativecommons.org/licenses/by/4.0/).
